# Optimized CRISPR guide RNA design for two high-fidelity Cas9 variants by deep learning

**DOI:** 10.1038/s41467-019-12281-8

**Published:** 2019-09-19

**Authors:** Daqi Wang, Chengdong Zhang, Bei Wang, Bin Li, Qiang Wang, Dong Liu, Hongyan Wang, Yan Zhou, Leming Shi, Feng Lan, Yongming Wang

**Affiliations:** 1 State Key Laboratory of Genetic Engineering, School of Life Sciences, Zhongshan Hospital, Fudan University, Shanghai, 200432 China; 2 Co-innovation Center of Neuroregeneration, Jiangsu Key Laboratory of Neuroregeneration, Nantong University, Nantong, 226001 China; 3 Hospital of Obstetrics and Gynecology, Fudan University, Shanghai, 200011 China; 4 Human Phenome Institute, Fudan University, Shanghai, 200438 China; 5 Beijing Anzhen Hospital, Beijing Institute of Heart Lung and Blood Vessel Disease, Capital Medical University, Beijing, 100029 China

**Keywords:** Bioinformatics, CRISPR-Cas9 genome editing, Machine learning

## Abstract

Highly specific Cas9 nucleases derived from SpCas9 are valuable tools for genome editing, but their wide applications are hampered by a lack of knowledge governing guide RNA (gRNA) activity. Here, we perform a genome-scale screen to measure gRNA activity for two highly specific SpCas9 variants (eSpCas9(1.1) and SpCas9-HF1) and wild-type SpCas9 (WT-SpCas9) in human cells, and obtain indel rates of over 50,000 gRNAs for each nuclease, covering ~20,000 genes. We evaluate the contribution of 1,031 features to gRNA activity and develope models for activity prediction. Our data reveals that a combination of RNN with important biological features outperforms other models for activity prediction. We further demonstrate that our model outperforms other popular gRNA design tools. Finally, we develop an online design tool DeepHF for the three Cas9 nucleases. The database, as well as the designer tool, is freely accessible via a web server, http://www.DeepHF.com/.

## Abstract (web summary)

Application of highly specific Cas9 variants can be restricted by the design of the guide RNA. Here the authors present DeepHF, a gRNA activity prediction tool built from genome-scale screens of 50,000 guides covering 20,000 genes.

## Introduction

The CRISPR/Cas9 system derived from *Streptococcus pyogenes* (*Sp*Cas9) is currently considered a state-of-the-art tool for genome editing, and is used in a wide variety of organisms and cell types^1–6^. Despite significant advances in our understanding of the CRISPR/Cas9 system, concerns remain over the potential for off-target effects, which enormously impede clinical application of the technology^7–14^. One solution to address off-target limitations is to engineer SpCas9 with higher specificities. In a search for better genome editing, two SpCas9 variants, enhanced SpCas9 (eSpCas9(1.1)) and Cas9-High Fidelity (SpCas9-HF1), have been first generated by hypothesis that amino acid substitutions predicted to weaken nonspecific interactions between a Cas9-RNA complex and its substrate DNA would reduce off-target cleavage in cells^15,16^. By analysis of crystal structures of Cas9 variants, Chen et al. have generated a new hyper-accurate Cas9 variant (HypaCas9)^17^. There are now three additional highly specific Cas9 nucleases that have been developed using mutational library or directed evolution strategies^18–20^.

The CRISPR/Cas9 system consists of a Cas9 nuclease and a 100 nucleotides guide RNA (gRNA), which form a Cas9-gRNA complex, recognizing a 20 nucleotides target sequence with an NGG downstream protospacer adjacent motif (PAM, N_20_NGG) and induces a site-specific double-strand break (DSB)^1–3^. The success of genome editing depends on the choice of the gRNA sequence. Some gRNAs are capable of disrupting almost every target allele in a population of cells, while others display no detectable activity. This has led to development of several gRNA design tools for wild-type SpCas9 (WT-SpCas9) by using various algorithms, including linear regression model^21^, penalized linear regression model^22^, support vector machine (SVM) model^23,24^, and gradient-boosted regression model^25^. Intriguingly, two groups have recently shown that a convolutional neural network (CNN), a class of deep learning, improves gRNA design^26,27^.

The extensive applications of highly specific SpCas9 variants have been limited by the lack of knowledge that governs gRNA activity. Existing evidence reveals that some highly active gRNAs for WT-SpCas9 are poorly active for the highly specific Cas9 variants^15,16,18^. A recent genome-wide activity profiling study in bacteria reveals the differences in the profile of important features between WT-SpCas9 and eSpCas9^28^. In this study, we perform a genome-scale screen in human cells to measure gRNA activity for WT-SpCas9, eSpCas9(1.1), and SpCas9-HF1 by using a high-throughput method and obtain an activity data set of over 50,000 gRNAs, covering ~20,000 genes. Finally, we develop a deep-learning-based online design tool for the three Cas9 nucleases.

## Results

### A mouse U6 (mU6) promoter expands genomic targeting sites

An optimal gRNA library design requires a large number of accessible genomic target sites. The gRNA transcription is commonly driven by human U6 (hU6) promoter that is believed to require guanine (G) as the first nucleotide of its transcript^1–3^. In the event that the first nucleotide is not a G, it is possible to replace the first nucleotide with a G or add an extra G to the 5′ end of gRNA, resulting in a gRNA–DNA mismatch at 5′ end. WT-SpCas9 can tolerate gRNA–DNA mismatches at 5′ end, so it can target any N_20_NGG sequence with hU6 promoter^1–3^. However, highly specific Cas9 nucleases such as eSpCas9(1.1) and SpCas9-HF1 are sensitive to gRNA–DNA mismatches at 5′ end^29^. They can only target GN_19_NGG sequence when the hU6 promoter is used, limiting target site selection.

**Fig. 1:**
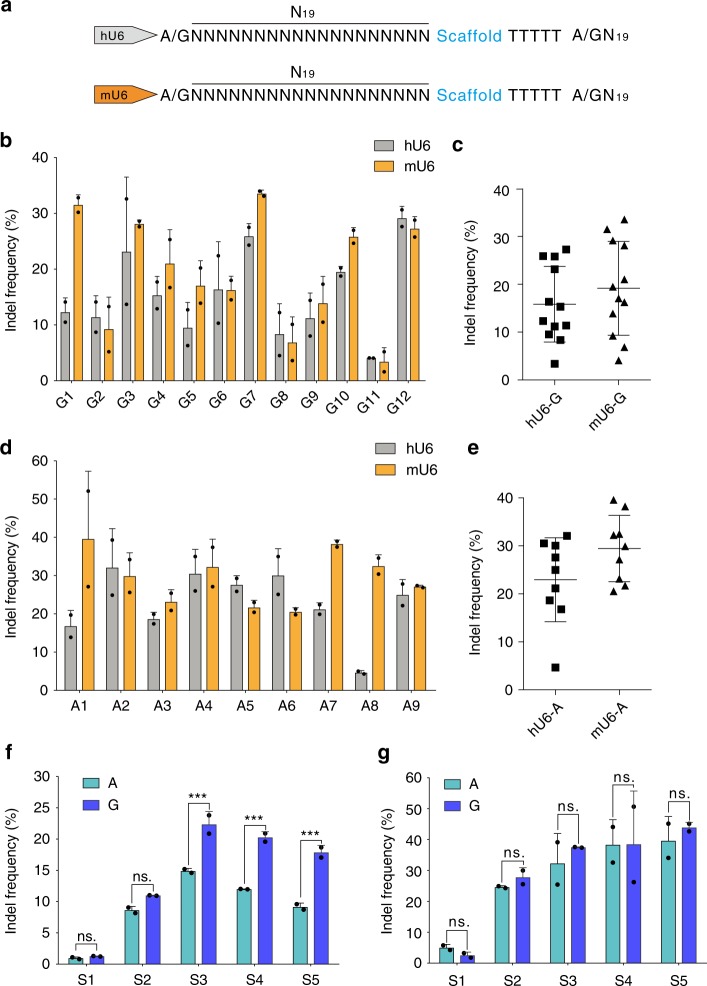
Both mU6 promoter and hU6 promoter enable to transcript gRNAs initiated with A or G for genome editing. **a** Schematic diagram of mU6 and hU6 comparison. These two promoters were used to transcript gRNAs initiated with A or G nucleotide. **b**, **c** Comparison of mU6 and hU6 promoters for genome editing with gRNAs initiated with G. The data are shown as mean ± s.d. (*n* = 2). **d**, **e** Comparison of mU6 promoter for genome editing with gRNAs initiated with A. The data are shown as mean ± s.d. (*n* = 2). **f** Comparison of the mU6 promoter for genome editing with gRNAs initiated with A or G three days post transfection. **g** Comparison of the mU6 promoter for genome editing with gRNAs initiated with A or G 5 days post transfection. The data are shown as mean ± s.d. *P* > 0.05; *P* < 0.05 by two-way ANOVA (*n* = 2). Source data are provided as a Source Data file

A previous study has shown that the mouse U6 (mU6) promoter can initiate either adenine (A) or G transcript^30^, which could potentially expand target selection. We compared the activity of mU6 promoter and hU6 promoter by transient expression of gRNAs for genome editing in WT-SpCas9-expressing HEK293T cells (Fig. 1a). The mU6 promoter showed activity similar to hU6 promoter for 12 tested gRNAs initiated with G (Fig. 1b, c, Table 1; Supplementary Fig. 1a). We tested nine gRNAs initiated with A but free of G at 1–4 nucleotides, avoiding functional truncated gRNAs transcribed from G (Table 1). To our surprise, both promoters could promote genome editing with gRNAs initiated with A (Fig. 1d, e; Supplementary Fig. 1b). Among all nine tested gRNAs, only one gRNA (A8) driven by hU6 displayed low efficiency. We tested additional nine gRNAs initiated with A, but contained G at 1–5 nucleotides. In this case, mU6 promoter showed generally higher activity than hU6 promoter (Supplementary Fig. 2a–c). Next, we compared the activity of mU6 promoter and hU6 promoter in HeLa cells, and they showed similar activity (Supplementary Fig. 3a–f). Furthermore, we compared the activity of mU6 promoter and hU6 promoter in a lentivirus vector, and they showed similar activity at two time points, day 3 and day 5, after transduction (Supplementary Fig. 4a, b). Our results are consistent with a very recent study that hU6 promoter can transcribe small RNAs initiated with A^31^.

**Table 1:** gRNAs used in Fig. 1

Name		Sequences
AN_19_	A1	ACCTTCACCTGGGCCAGGGA
	A2	ACCCACGGCTACAAAGCGCA
	A3	ACTACAGAAAGCCAAACAAA
	A4	ACCTGGCCTACTGTACACCG
	A5	AACCGCTCTATGTCCAGCTG
	A6	ATCTGGACTTTCACAATCAG
	A7	ACCTTGGCTTGGCTTTGCTG
	A8	ACACAGTGGGCCAGAGAGAA
	A9	ATTCACAGAAGGGGATGGCA
C/TN_19_	S1	a/gTCTTCTTCTGCTCGGACTC
	S2	a/gTCCCCATTGGCCTGCTTCG
	S3	a/gCCAGCTTGGGCCCACGCAG
	S4	a/gCACCTCCAATGACTAGGGT
	S5	a/gAAACGGCAGAAGCTGGAGG
GN_19_	G1	GACACAGTGGGCCAGAGAGA
	G2	GTAGCCTCAGTCTTCCCATC
	G3	GCTCCCATCACATCAACCGG
	G4	GTACAAACGGCAGAAGCTGG
	G5	GAGGCCCCCAGAGCAGCCAC
	G6	GCACAGATGAGAAACTCAGG
	G7	GAGTCCGAGCAGAAGAAGAA
	G8	GGGTTAGGGGCCCCAGGCCG
	G9	GTCACCTCCAATGACTAGGG
	G10	GCCTCCCCAAAGCCTGGCCA
	G11	GCCCCGGGCTTCAAGCCCTG
	G12	GCTTGTCCCTCTGTCAATGG

Next, we tested the activity of the mU6 promoter for genome editing with gRNAs initiated with C or T, but changed them to A or G (Table 1). After 3 days of genome editing, gRNAs initiated with G showed higher activity for three of five tested gRNAs (Fig. 1f; Supplementary Fig. 5a), but the difference was eliminated after 5 days (Fig. 1g; Supplementary Fig. 5b). The mU6 promoter was chosen in the following study.

### A strategy for high-throughput test of gRNA activity

**Fig. 2:**
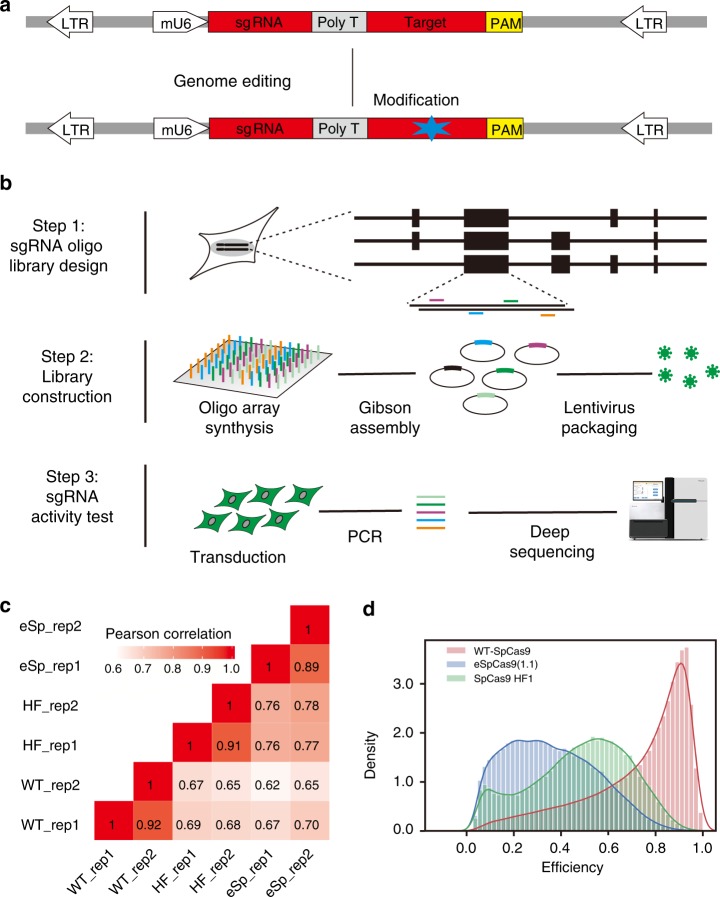
A guide RNA–target pair strategy for high-throughput screen of gRNA activity in human cells. **a** Schematic diagram of the guide RNA–target pair strategy for gRNA activity test. A lentiviral vector contains a mU6 promoter, and a guide RNA–target pair. The vector was used to transduce cells expressing Cas9 nucleases. Indels would be induced at integrated targets by the corresponding gRNAs. **b** Schematic diagram of design and high-throughput screen of a gRNA library. A library of 80,263 guide RNA–target pairs was designed and synthesized by microarray. Oligonucleotides were PCR-amplified and cloned into lentiviral vectors by Gibson assembly. The library was packed into viruses and transduced into cells expressing Cas9 nucleases for genome editing. The integrated target sites were PCR-amplified for deep-sequencing analysis. **c** The Pearson correlation of indel frequency among different experiment repeats. **d** The distribution of gRNA activity for the three Cas9 nucleases

A recent study has shown that a guide RNA–target pair strategy enables high-throughput test of gRNA activity for Cpf1^32^. In this strategy, the synthesized guide RNA–target sequences are delivered into Cas9-expressing cells by lentiviruses (Fig. 2a). After genome editing, the target sequences are PCR-amplified for deep sequencing, allowing direct measurement of insertion/deletion (indel) rates induced by Cas9 nucleases. An additional advantage is that lentiviruses preferentially integrate into transcriptionally active regions which are much more accessible for the CRISPR/Cas9 machinery^32–34^, minimizing the influence of genome editing by chromatin accessibility. Therefore, the data set obtained by this strategy provides the opportunity to elucidate the inherent activity of gRNAs based exclusively on their sequence features.

Doench et al. developed an online tool that allows designing gRNAs for gene knockout with WT-SpCas9^25^. This tool scans a whole gene-coding sequence and ranks all gRNAs based on activity and off-target effects. We used this tool to design gRNAs for the library screening. Four top-ranked gRNAs initiated with either A or G were selected for each gene (Fig. 2b). We also designed gRNAs targeting microRNAs. As the lengths of microRNA coding sequences are much shorter than the gene-coding regions, we typically designed three gRNAs for each microRNA. A total of 80,263 oligonucleotides that contain gRNAs and corresponding target sequences (75,312 gRNAs for 19,037 coding genes; 4951 gRNAs for 1549 microRNAs) were synthesized by microarray (Supplementary Data 1). The oligonucleotides were PCR amplified and cloned into the lentivirus vectors via Gibson assembly. Analysis of the plasmid library by deep sequencing revealed that the error rate (A read contains any mutation was considered as an error) induced by oligonucleotide synthesis or PCR amplification at guide RNA–target sequence region was 36.5%. This plasmid library was used in the following pooled screening experiments to profile gRNA activity for WT-SpCas9, eSpCas9(1.1), and SpCas9-HF1.

The library was packaged into lentiviruses and transduced into HEK293T cells expressing WT-SpCas9, eSpCas9(1.1), or SpCas9-HF1 at an MOI of 0.3. After 5 days of genome editing, genomic DNA was extracted, and the integrated target regions were PCR amplified for deep sequencing (Fig. 2b). The mutations at guide RNA–target sequence regions can be induced either by Cas9 nucleases or by library construction. If a mutation can be found in the original library, it was considered as a mutation induced by library construction and excluded from indel analysis. Indels could be detected by deep sequencing at the integrated target sites (Supplementary Fig. 6a). We obtained valid gRNA indel rates (reads number > 100) of 55,604 (covering 20,211 genes), 58,167 (covering 20,315 genes), and 56,888 (covering 20,270 genes) for WT-SpCas9, eSpCas9(1.1), and SpCas9-HF1, respectively (Supplementary Data 2). To the best of our knowledge, this is the largest gRNA on-target activity sets reported so far in mammalian cells.

The screening assay was experimentally repeated twice, and two independent replicates showed a high level of correlation for indel rate (*R* = 0.92 for WT-SpCas9; *R* = 0.89 for eSpCas9(1.1); *R* = 0.91 for SpCas9-HF1, Fig. 2c). The indel rate of individual gRNAs also has a strong correlation among three Cas9 nucleases (Fig. 2c), indicating that some sequence features are favored for these three Cas9 nucleases. The distribution of gRNA activities varied remarkably, from no activity to 100% indel rates for these three Cas9 nucleases (Fig. 2d). WT-SpCas9 showed higher efficiency of editing than eSpCas9(1.1) and SpCas9-HF1 in our screening (Supplementary Fig. 6b). Since these were single-cell-derived clones that could influence efficiencies, we selected five gRNAs and transfected them together with individual Cas9 nuclease into cells. WT-SpCas9 and SpCas9-HF1 showed similar activity, but eSpCas9(1.1) showed lower activity at day 3 (Supplementary Fig. 6c), consistent with previous studies^15,16^.

It has been reported that residual plasmid DNA from viral packaging procedures can contaminate transduced cells^35^, resulting in potential inaccuracies in measurement of gRNA activity. We designed a pair of primers specific for the backbone of plasmids to detect residual plasmids, and a pair of primers specific for the lentivirus genomic DNA to detect both residual plasmids and lentiviruses integrated into the genome (Supplementary Fig. 7a). These two pairs of primers displayed similar efficiency of amplification when plasmid DNA was used as templates (Supplementary Fig. 7b). The residual plasmid DNA could be detected in both unconcentrated viruses and concentrated viruses during virus packaging (Supplementary Fig. 7b). We transduced HEK293T cells and extracted genomic DNA at day 1 and day 5 after transduction, respectively. The residual plasmid DNA could be detected in both samples, but the PCR bands were very weak at day 5 with primer specific for backbone, indicating that the residual plasmid degraded over time (Supplementary Fig. 7b). In contrast, very strong bands could be detected with primers specific for the viruses at day 5, indicating that the more and more lentiviruses integrated into genome. These results suggested that the residual plasmid DNA only had minimal influence on the library screen.

### Sequence features associated with gRNA activity

Characterization of sequence features associated with gRNA activity is crucial for the development of gRNA design tools. This large-scale data set generated here allows us to better evaluate which features contributed most to gRNA activity. Algorithms including gradient-boosted regression trees and lasso regression have been used to assess feature importance^36^. However, gradient-boosted regression trees provide Gini importance scores that only reflect the absolute value of feature contribution, causing the loss of information regarding the direction of the effect; lasso regression displays poor descriptive ability. Fortunately, a recently developed algorithm SHAP (SHapley Additive exPlanation), a unified approach to explain the output of any machine-learning model, can potentially address these limitations^37^.

**Fig. 3:**
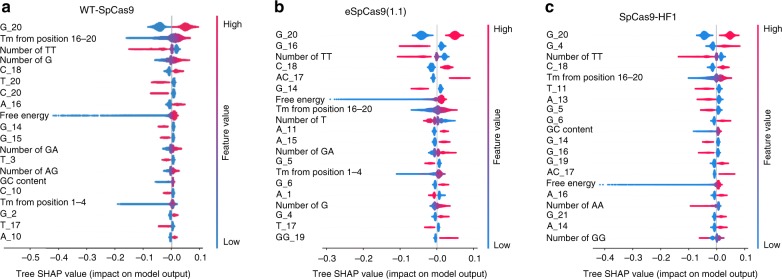
Analysis of feature importance associated with gRNA activity by Tree SHAP. **a**–**c** Top 20% important features identified by Tree SHAP for WT-SpCas9, eSpCas9(1.1), and SpCas9-HF1, respectively. The nucleotides, as well as their position, were shown on the left. GG_19 means GG dimer start at position 19. *T*_m_ means melting temperature

We connected XGBoost with SHAP (called Tree SHAP) to evaluate the importance of 1031 features, including features identified by Doench and Wong et al.^25,38^, and all of the position accessibilities of gRNA secondary structure features (Supplementary Data 3, 4). Overall, the predicted scores were strongly influenced by position-dependent nucleotide composition for three Cas9 nucleases (Fig. 3a–c; Supplementary Data 4). The most favored nucleotide was G at position 20 (G_20), the nucleotide immediately adjacent to the PAM sequence. Other important features overlapped in the top 20 for three nucleases are melting temperature (*T*_m_, favored), number of TT dimers (disfavored), C_18 (favored), self-folding free energy (favored), and G_14 (disfavored).

**Fig. 4:**
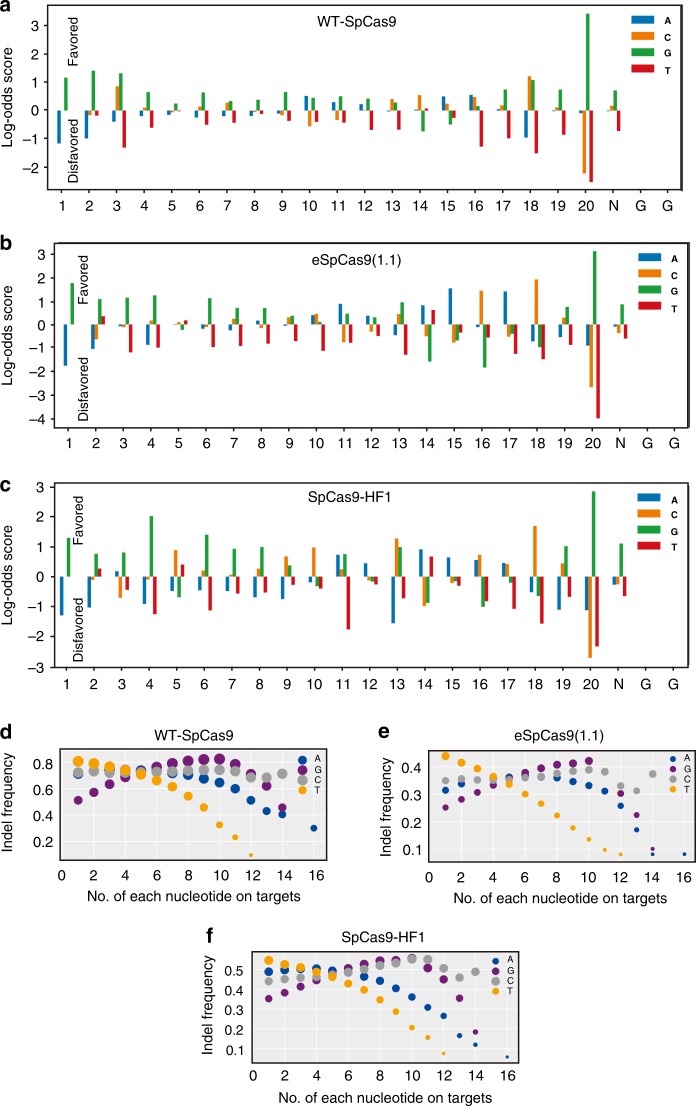
Influence of nucleotide composition on gRNA activity. **a**–**c** The position-dependent nucleotide composition of the highest 25% active gRNAs versus lowest 25% active gRNAs. Bars showed log-odds scores of nucleotide frequency for each position. The numbers below indicated the position of the nucleotides on-target DNA. **d**–**f** The association of each nucleotide number with gRNA activity. The size of the circles indicated indel frequency

We next evaluated position-dependent nucleotide composition of the highest 25% active gRNAs versus lowest 25% active gRNAs. The results revealed that G was generally favored, and T was generally disfavored (Fig. 4a–c; Supplementary Data 5). G_20 was strongly favored for all three Cas9 nucleases, consistent with Tree SHAP analysis. The differences of nucleotide preference between WT-SpCas9 and SpCas9 variants were also observed. The differences of favored nucleotide at position 3 (C/G vs G), 9 (G vs C/G), 10 (A/G vs A/C), 14 (C vs A/T), 16 (A/C vs C), 17 (G vs A), and 18 (C/G vs C) were observed between WT-SpCas9 and eSpCas9(1.1). The differences of favored nucleotide at position 3 (C/G vs G), 5 (G vs C/T), 7 (C/G vs G), 9 (G vs C/G), 10 (A/G vs C), 12 (A/G vs A), 14 (C vs A/T), 17 (G vs A/C), and 18 (C/G vs C) were observed between WT-SpCas9 and SpCas9-HF1. In addition, we analyzed position-independent nucleotide composition with top 20% active gRNAs (Fig. 4d–f). G (favored) and T (disfavored) content strongly influenced gRNA activity, while A and C content mildly influenced gRNA activity for three Cas9 nucleases, consistent with Tree SHAP values.

### Performance of conventional algorithms

**Fig. 5:**
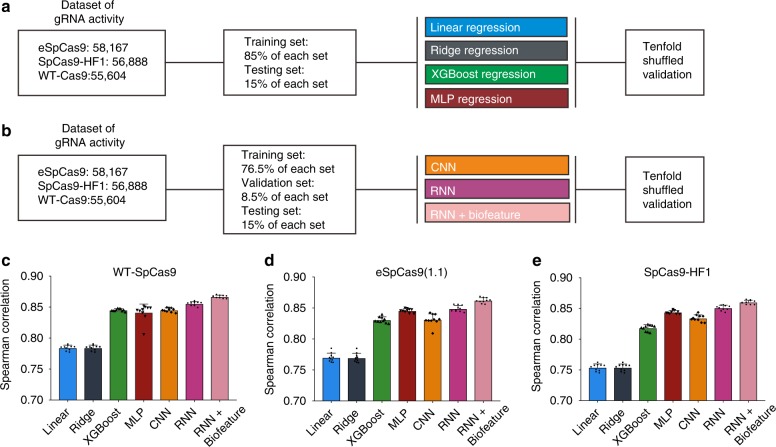
Performance of different algorithms for gRNA activity prediction. **a** Schematic of data set and conventional algorithms. Four conventional algorithms including linear regression, ridge regression, XGBoost regression, and MLP were constructed, respectively. In all, 85% of the relevant data set was used as the training set, and the reserved 15% of the data set in each set as the test set to measure the generalization ability of each model to predict unseen data. **b** Schematic of the data set and deep-learning algorithms. Deep-learning algorithms including CNN, RNN, and RNN + biofeature were constructed, respectively. In total, 76.5% of the relevant data set was used as the training set, 8.5% of the data set in each set was used as the validation set, and the reserved 15% of the data set in each set was used as the test set to measure the generalization ability of each model to predict unseen data. **c**–**e** Performance of different algorithms for gRNA activity prediction revealed by Spearman correlation for WT-SpCas9, eSpCas9(1.1), and SpCas9-HF1, respectively. The bar plot shows the mean ± s.d. for the Spearman correlation coefficient between predicted and measured gRNA activity scores (*n* = 10)

In addition to generating data sets of gRNA activity, another goal of this work was to develop prediction tools for gRNA design. We evaluated the performance of four conventional gRNA activity prediction algorithms, including Linear regression, L2-regularized linear regression (Ridge regression), XGBoost regression, and multilayer perceptron (MLP) models with data sets generated in this study. To prevent over-fitting, we randomly separated the data set into two subgroups with 85% of the data used as the training data set to train the models, and the remaining 15% held out used to test the generalization capacity of the trained models (Fig. 5a). To achieve optimal performance, the features with high Tree SHAP values were modeled in the algorithms (Supplementary Data 4).

Of four algorithms tested here, MLP is the most predictive, with Spearman correlation coefficients of 0.8416, 0.8457, and 0.8440 for WT-SpCas9, eSpCas9(1.1), and SpCas9-HF1, respectively (Fig. 5c–e; Supplementary Data 6–8). XGBoost is the second most predictive, with Spearman correlation coefficients of 0.8454, 0.8310, and 0.8184 for WT-SpCas9, eSpCas9(1.1), and SpCas9-HF1, respectively. Linear regression and ridge regression also performed well, but with relatively lower correlation score. We also tried lasso regression and SVM regression. But the penalty coefficient for lasso regression is nearly zero, which made it equivalent to linear model. SVM regression failed to finish the benchmarking on the current scale of data set within a reasonable time (3 weeks). They were dropped in the final comparison.

### Performance of deep-learning algorithms

Recent studies have shown that two deep-learning-based algorithms, convolutional neural network (CNN) and recurrent neural network (RNN), are powerful tools for DNA/protein sequence-related analysis^39–43^. They could obtain useful features from raw DNA/protein sequence automatically without requirement of feature engineering. CNN has been used to predict gRNA activity for Cpf1 and WT-SpCas9^26,27^, while RNN has not been used for gRNA activity prediction so far. We trained both CNN and RNN for gRNA activity prediction. To prevent over-fitting, we randomly separated the data set into three subgroups with 76.5% of the data used as the training data set to train the models, 8.5% used as validation data set, and the remaining 15% held out used to test the generalization capacity of the trained models (Fig. 5b).

RNN outperformed CNN and other algorithms for gRNA activity prediction with Spearman correlation coefficients of 0.8555, 0.8491, and 0.8512 for WT-SpCas9, eSpCas9(1.1), and SpCas9-HF1, respectively (Fig. 5c–e; Supplementary Data 6–8). CNN obtained similar performance to XGBoost with Spearman correlation coefficients of 0.8455, 0.8313, and 0.8343 for WT-SpCas9, eSpCas9(1.1), and SpCas9-HF1, respectively.

### An integrated model improves predictive power

**Fig. 6:**
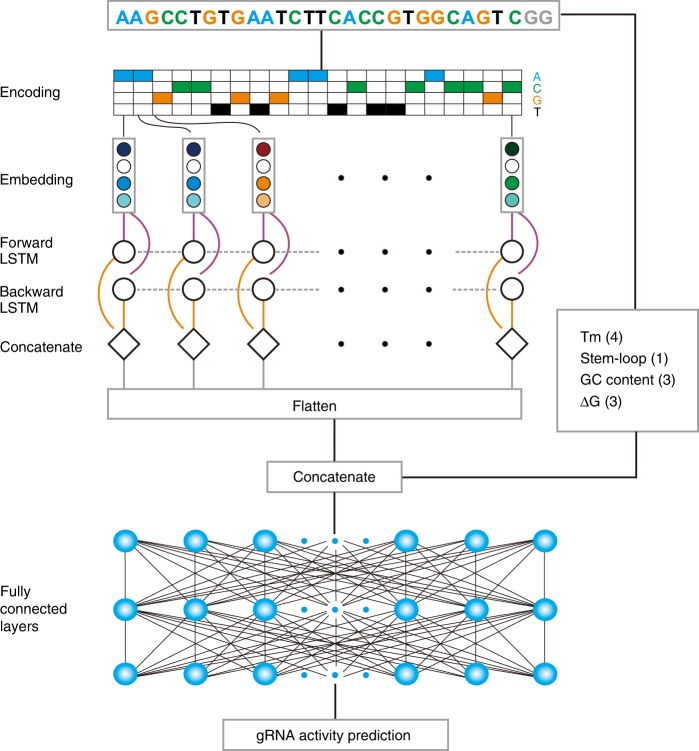
DeepHF model for gRNA activity prediction. The original gRNA sequence is first encoded and then embedded to get a new representation. This new representation is further processed by a BiLSTM to get the final representation which is then concatenated with hand-crafted features to serve as the input of the fully connected layer to be nonlinearly transformed. Finally, a linear transformation is performed to get the prediction score

The backbone of the deep-learning algorithms (CNN or RNN) can only exploit k-mer composition or its dependencies^39,44^. Recent studies on protein-related prediction have shown that the prediction ability of deep-learning models could be boosted by addition of other features, such as molecular weight, hydrophobicity, and absolute charge, which could not be automatically obtained by deep-learning models^45,46^. In our work, indirect sequence features including position accessibilities of secondary structure, stem–loop of secondary structure, melting temperature, and GC content are strongly associated with gRNA activity (Supplementary Data 4), but they could not be obtained by deep learning. Considering that the RNN model achieved the best performance of all the algorithms, we thus combined these biological features with RNN for gRNA activity prediction. Interestingly, addition of these features to RNN increased prediction power, with Spearman correlation coefficients of 0.8670, 0.8624, and 0.8603 for WT-SpCas9, eSpCas9(1.1), and SpCas9-HF1, respectively (Fig. 5c–e). Therefore, RNN integrated with biological features (hereafter referred to as RNN + Biofeature) was used as the final model for gRNA activity prediction (Fig. 6).

To further test the performance of seven models used in this study, we generated a list of gRNA indel rates for endogenous sites (85 sites for WT-SpCas9, 81 sites for eSpCas9(1.1), and 82 sites for SpCas9-HF1) (Supplementary Data 9). All seven models worked considerably well, but linear regression and ridge regression were less predictive based on Spearman correlation metrics (Supplementary Figs. 8–10). Due to the limited data set here, we could not conclude which algorithm was statistically better than other algorithms on endogenous sites. We also investigated the correlation of the indel frequency at the synthetic targets with that at the corresponding endogenous targets. The Spearman correlation is 0.722, 0.767, and 0.730 for WT-SpCas9, eSpCas9(1.1), and SpCas9-HF1, respectively (Supplementary Fig. 11a–c).

### Comparison of the RNN + Biofeature model with existing models

**Fig. 7:**
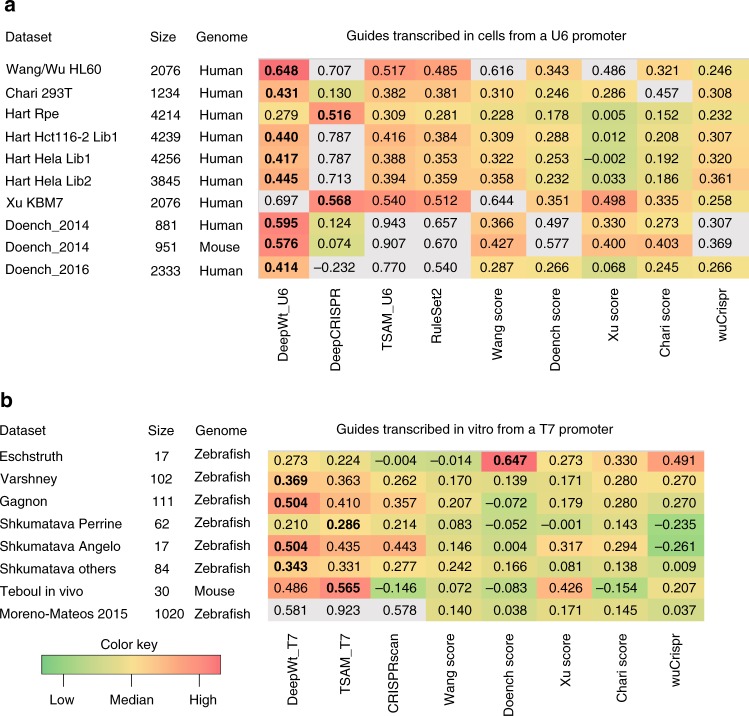
Heatmap of Spearman rank correlation coefficients between efficiency scores and data sets. **a** gRNAs transcribed in cells from a U6 promoter. **b** gRNAs transcribed in vitro from a T7 promoter. Cell type, the number of gRNAs, and species are shown on the left. Scores are shown along the horizontal axis, data sets on the vertical. Correlations of an algorithm against its own training data set are shown in gray as they are likely to be overestimated due to over-fitting. The highest scores are in bold

There are several publicly available data sets of gRNA efficiency for WT-SpCas9, which allows us to compare the performance of the RNN + Biofeature model for WT-SpCas9 (hereafter referred to as DeepWt) with existing prediction models. We tested DeepWt against 18 endogenous data sets collected by Haeussler et al.^47^, and obtained Spearman correlation coefficients varied from 0.129 to 0.594 (Supplementary Data 10). It has been reported that the prediction model strongly depends on whether the gRNA is expressed from a U6 promoter in cells or from a T7 promoter in vitro^47,48^. Therefore, the transfer learning strategy was used to improve the prediction ability of our model under different expression conditions. For U6 promoter expression, the performance was improved by fine-tuning of the last hidden layer of DeepWt with XuKBM data set (Supplementary Data 10). Our final optimized model, named DeepWt_U6, outperformed other seven popular gRNA design tools (Fig. 7a). Notably, since our gRNA library was designed, in part, by RuleSet2 which was developed by Doench data sets, this comparison may had a bias for DeepWt_U6 on Doench datasets. For T7 promoter expression, we developed another model named DeepWt_T7 by fine-tuning of RNN + biofeature algorithm with Moreno-Mateos2015 data set. This model outperformed other models for design of gRNAs expressed in vitro (Fig. 7b).

It has been reported that integration of target site accessibility metrics into the model could improve prediction power^26,27^. For WT-SpCas9, we fine-tuned the DeepWt_U6 model with DNase I data of KBM-7 cell line obtained from ENCODE, resulting in the DeepWt_Chromatin model. Wang/Xu HL60 and Hart Hct116-2 Lib 1 data sets as well as the corresponding DNase I data were used to test the performance of DeepWt_ Chromatin. However, the Spearman correlation scores were not improved (Supplementary Data 11). We also retrieved DNase I data of HEK293T cells from ENCODE database and tested whether integration of metrics into the models could improve prediction power for eSpCas9(1.1) and SpCas9-HF1. The DNase I data were processed following the method described by Kim et al.^26^. However, incorporation of these data could not significantly increase the prediction ability (tenfold shuffled validation) for eSpCas9(1.1) and SpCas9-HF1 (Supplementary Data 12).

### Nucleotide contributions revealed by Deep SHAP

In addition to the prediction accuracy, we are also interested in understanding the mechanisms of the deep-learning model. Lundberg and Lee^49^ developed an algorithm called Deep SHAP, which is a high-speed approximation algorithm for SHAP values in deep-learning models. We used Deep SHAP to estimate the position-dependent nucleotide contribution to the deep-learning model. The contribution of each position-dependent nucleotide to gRNA activity was computed from the average value of that position across all the training gRNAs. To make the contribution of nucleotides comparable among WT-SpCas9, eSpCas9(1.1), and SpCas9-HF1, Deep SHAP values were rescaled by the Z-score (i.e., standardization). The nucleotide with a Z-score above 1 or below −1 was considered to have a significant contribution to gRNA activity (Supplementary Fig. 12a).

There were 16, 21, and 26 significant nucleotides in WT-SpCas9, eSpCas9(1.1), and SpCas9-HF1, respectively. The result revealed that G typically had a positive contribution and T typically had a negative contribution, in agreement with previous observation that Cas9 preferentially binds gRNAs containing purines but not pyrimidines^50^. In addition, multiple Ts in the spacer caused low gRNA expression^51^. We found that most of the significant nucleotides had the same direction of contribution to gRNA activity in all three Cas9 nucleases. Consistent with several previous reports^38,52^, the most influential was the nucleotides at position 20, where G_20 had a strong positive contribution and C_20/T_20 had a strong negative contribution. Compared with WT-SpCas9, eSpCas9(1.1)-specific motifs included A_15 (favored), A_17 (favored), G_6-8 (favored), G_14 (disfavored), G_16 (disfavored), T_6 (disfavored), T_11-12 (disfavored); SpCas9-HF1-specific motifs included A_11 (favored), A_13 (disfavored), A_14-15 (favored), A_19-20 (disfavored), G_6-7 (favored), G_14 (disfavored), G_16 (disfavored), T_4 (disfavored), T_6 (disfavored), and T_11 (disfavored) (Supplementary Fig. 12a).

In addition, the difference of Z-score between Cas9 variants and WT-SpCas9 was calculated to assess changes in nucleotide contributions. The difference above 1 or below −1 was considered to have a significant change (Supplementary Fig. 12b). Several differences in the contribution direction of the significant nucleotides were observed. Specifically, A_13 contributed negatively to SpCas9-HF1 but not to WT-SpCas9, G_17 contributed positively to WT-SpCas9 but not to eSpCas9(1.1) and HF1-SpCas9, G_18 contributed positively to WT-SpCas9 but contributed negatively to eSpCas9(1.1) (also to HF1-SpCas9, but not significant).

The contribution of repetitive nucleotides to gRNA activities were also investigated based on the sum of Deep SHAP values. For WT-SpCas9, previous study has shown that a stretch of adjacent identical nucleotides (repetitive nucleotides, such as GGGG or TTTT) could be associated with poor efficiency of a sgRNA^38^. However, they did not consider position effects. Therefore, we calculated the average Deep SHAP values of the four repeat nucleotides including AAAA, CCCC, GGGG, and TTTT from position 1 to 18 (Supplementary Data 13). Our results demonstrated that the repetitive nucleotides generally decreased indel efficiency, consistent with Wong et al.’s study^38^. However, the positive contribution of repeat nucleotides at some positions were observed, including GGGG starting from position 1–5 and 16–18, AAAA starting from position 14, CCCC starting from position 1–2 and 15–18 (Supplementary Data 13). The positive contribution of repeat nucleotides at some positions was also observed for eSpCas9 (1.1) and SpCas9-HF1. For example, AAAA starting from position 14–15 contributed positively to gRNA activities for both Cas9 nucleases (Supplementary Data 13).

### The correlation of indel frequency to phenotype

In this study, we used indel rate as the gRNA activity label, which is not equal to real gene knockout efficiency. We tested the correlation between indel frequency and actual gene disruption with a protein-based assay. We designed a total of nine gRNAs targeting SIRT1, SIRT2, and SIRT6 with three gRNAs for each. The gRNAs and Cas9 nucleases were introduced into HEK293T cells with an episomal vector which allowed long-term genome editing^5^. The indel frequency and protein expression was analyzed at day 9 after transfection. The results revealed that indel frequencies had a good correlation to protein expression (*r* = 0.82; Supplementary Fig. 13a, b). In addition, the correlation between indel frequency and actual gene disruption was tested by a luciferase reporter assay. We designed a total of 11 gRNAs targeting luciferase gene. Five days after genome editing, indel frequency and luciferase activity was analyzed. The results revealed that indel frequencies had a good correlation to luciferase activity (*r* = 0.70, Supplementary Fig. 13c–e).

### The correlation between on-target and off-target efficiency

The goal of this work is to design gRNAs with better on-target activity, but such gRNAs may be greater tolerance for mismatches and thus induce higher off-target mutations. To test this hypothesis, we selected three sgRNAs with different activity and designed guide–target pairs with double mismatches (Supplementary Fig. 14a). Off-target cleavage occurred efficiently with target sequences containing mismatches at position 1–8 for WT-SpCas9 (Supplementary Fig. 14b). As expected, higher on-target activity generally led to higher off-target activity. For eSpCas9(1.1) and SpCas9-HF1, however, the off-target cleavage was at background level except mismatches at position 7–8, where site1-gRNA displayed high off-target cleavage (Supplementary Fig. 14b). Similar mismatch tolerance was also observed by Slaymaker et al.^15^. Site2-gRNA had comparable activity to site1-gRNA, but its off-target cleavage was at the background level, indicating that mismatch tolerance depends on gRNA sequences.

### Online service

We finally developed an online tool called DeepHF (Deep learning for High-Fidelity Cas9) based on RNN + biofeature model for gRNA design for WT-SpCas9, eSpCas9(1.1), and SpCas9-HF1. The online tool contains three functional modules, namely prediction module, verified gRNAs module, and design module. Prediction module allows users to get predicted activities for all gRNAs with an input DNA sequence. Verified gRNAs module provides all gRNA indel rates generated in this study. Design module provides gRNAs that are suitable for gene knockout with eSpCas9(1.1) and SpCas9-HF1 in human cells. In this module, gRNAs were chosen from common transcripts of each gene (Genome Reference Consortium Human Build 38). The off-target information (1–3 mismatches considered as off-target) and targeting location (whether in the 5–65% of coding sequence) were also annotated. Users can obtain the predesigned gRNAs by inputting a gene ID or a gene symbol. The website is freely available at http://www.DeepHF.com/.

## Discussion

Broader application of highly specific Cas9 nucleases has been hampered by lack of knowledge for gRNA design. Our study filled the gap by generating a database of over 50,000 gRNAs covering ~20,000 human genes for eSpCas9(1.1) and SpCas9-HF1. Users can pick efficient gRNAs from the database for gene knockout. In addition, we have shown here that the Tree SHAP algorithm is a powerful tool for evaluation of feature importance. Based on large data set and important features, we optimized seven models for gRNA activity prediction. Importantly, we have demonstrated that RNN + biofeature is to the best of our knowledge, a state-of-the-art model for activity prediction for the three Cas9 nucleases. These useful clues will facilitate the development of optimal computer models for gRNA design for other Cas9 nucleases. We finally developed an online tool for gRNA design for WT-SpCas9, eSpCas9(1.1), and SpCas9-HF1. Taken together, our study will facilitate application of highly specific cas9 nucleases for genome editing.

## Methods

### Cell culture and transfection

HEK293T and HeLa cells (ATCC) were maintained in the Dulbecco’s Modified Eagle Medium (DMEM) supplemented with 10% FBS (Gibco), 100 U/ml penicillin, and 100 mg/ml streptomycin at 37 °C and 5% CO_2_. For transfection, HEK293T or HeLa cells were plated into 24-well or 12-well plates, DNA mixed with Lipofectamine 2000 (Life Technologies) in Opti-MEM according to the manufacturer’s instructions. HEK293T and HeLa cells were used to test the indel efficiency and tested negative for mycoplasma; and cells identities were validated by STR profiling (ATCC).

### Plasmid construction

To compare the activity of hU6 and mU6 promoters for genome editing, plasmids expressing gRNAs were modified from epiCRISPR vectors^5^. Briefly, the EF1-Cas9 fragment (AgeI-NheI) on epiCRISPR was replaced by a CMV promoter, resulting in epiCRISPR-hU6-gRNA vector; the hU6 promoter was replaced by an mU6 promoter using Gibson Assembly (NEB), resulting in epiCRISPR-mU6-gRNA vector. The gRNA oligonucleotide pairs were annealed and cloned into BspQI sites.

To compare the activity of hU6 and mU6 promoters in lentivirus system, we replaced the hU6 promoter (linearization by PCR) on lentiGuide-Puro vector (addgene # 52963) with mU6 promoter by using Gibson Assembly (NEB), resulting in lentiGuide-Puro-mU6. The gRNA oligonucleotide pairs were annealed and cloned into BsmBI sites.

Lenti-gRNA–target vector constructed as follows. First, we replaced the EF1a promoter (AsiSI-XbaI site) on pCDH_EF1_MCS_T2A_Puro vector (SBI, Palo Alto, CA, USA) with CMV promoter, resulting in pCDH_CMV_MCS_T2A_Puro (pCP); second, the mU6 promoter was cloned into XbaI-BstBI site of pCP, resulting in pCP-mU6 (pCmP); third, the eGFP coding sequence was cloned into the BstBI-BamHI site of pCmP, resulting in pCmP-eGFP (pCmeP); finally, the “Filler” fragment was PCR amplified from plentiCRISPR^53^ and cloned into pCmeP (BstBI-XhoI site), resulting Lenti-gRNA–target vector (Supplementary Fig. 15).

### Lentivirus production

For individual sgRNA packaging, HEK293T cells were seeded at ~40% confluency in a six-well dish the day before transfection. For each well, 1.2 μg of gRNA expressing plasmid, 0.9 μg of psPAX2, and 0.3 μg of pMD2.G (Addgene) were transfected using 5 μl of Lipofectamine 2000 (Life Technologies). Media was changed 8 h after transfection. After 48 h, virus supernatants were collected and filtered with a 0.45 μm polyvinylidene fluoride filter and stored at −80 °C.

For library packaging, HEK293T cells were seeded at ~40% confluency in five 10-cm dishes the day before transfection. For each dish, 12 μg of plasmid library, 9 μg of psPAX2, and 3 μg of pMD2.G (Addgene) were transfected with 60 μl of Lipofectamine 2000 (Life Technologies). Virus was harvested twice at 48 h and 72 h post transfection. The virus was concentrated using PEG8000 (no. LV810A-1, SBI, Palo Alto, CA) dissolved in PBS and stored at −80 °C.

### Cell line expressing Cas9 generation

We introduced mutations on Cas9 nucleases on the LentiCas9-blast^54^ plasmid and generated plasmid expressing eSpCas9 (1.1) and SpCas9-HF1, respectively. LentiCas9-blast plasmid was packaged and transduced into HEK293T and HeLa cells. After 24 h, cells were selected with 10 μg/ml of blasticidin for 14 days. Single cells were sorted into 96-well plates for colony formation. Western blot with Anti-flag antibody was performed to screen cell clones with high levels of Cas9 expression (Supplementary Fig. 16). Western blots were incubated overnight at 4 °C with anti-FLAG antibody (14793 S, CST) at 1:1000 dilution.

### The gRNA library design

The gRNAs targeting microRNAs were designed using an in-house Python script. The gRNAs were selected if they satisfied the following criteria: (1) initiated with either A or G; (2) without four consecutive thymines (T). For each microRNA, we chose four gRNAs if possible or all of them if the number of gRNAs was less than four. Consequently, we designed a total of 4951 gRNAs covering 1549 human microRNAs.

The gRNAs targeting coding sequences in the library were designed by an online tool (https://portals.broadinstitute.org/gpp/public/analysis-tools/sgrna-design) developed by Doench et al.^25^. We selected ~four top-ranked gRNAs for each gene. A total of 75,312 gRNAs covering 19,037 human coding genes was designed. DNA oligonucleotides were synthesized and purchased from CustomArray, Inc. (Bothell, WA). The sequences of the oligonucleotides are listed in Supplementary Data 1.

### The gRNA library construction

Full-length oligonucleotides were PCR-amplified using Q5 High-Fidelity 2X Master Mix (NEB), size-selected using a 2% agarose EGel EX (Life Technologies, Qiagen), and purified using MinElute Gel Extraction Kit (Qiagen). PCR products were cloned into Lentiviral vector (Supplementary Fig. 15) by Gibson Assembly (NEB) and purified with Agencourt AMPure XP SPRI beads according to the manufacturer’s instructions (Beckman Coulter). The Gibson Assembly products were electroporated into MegaX DH10B^TM^ T1^R^ Electrocomp^TM^ Cells (Invitrogen) according to the manufacturer’s protocol using a GenePulser (BioRad) and grown at 32 °C, 225 rpm for 16 h. The bacterial clones covered the library at least 25-fold. The plasmid DNA was extracted from bacterial cells using Endotoxin-Free Plasmid Maxiprep (Qiagen).

### Screening experiments in human cells

HEK293T cells were plated into 15 cm dish at ~30% confluence. After 24 h, cells were infected with gRNA library with at least 1000-fold coverage of each gRNAs. After 24 h, the cells were cultured in the media supplemented with 2 µg/ml of puromycin for 4 days. Cells were harvested and the genomic DNA was isolated using Blood & Cell Culture DNA Kits (Qiagen) following the manufacturer’s instructions. The integrated region containing the gRNA coding sequences and target sequences were PCR amplified using primers Deep-seq-library-F/R with Q5 High-Fidelity 2X Master Mix (NEB). We performed 66 PCR reactions using 10 µg of genomic DNA as a template per reaction for deep-sequencing analysis; we took eight independent PCR reactions using 20 ng of plasmid DNA as a template per reaction. The PCR conditions: 98 °C for 2 min, 25 cycles of 98 °C for 7 s, 67 °C for 15 s, and 72 °C for 10 s, and the final extension, 72 °C for 2 min. The PCR products were mixed and purified using the Gel Extraction Kit (Qiagen). The purified products were sequenced on Illumina HiSeq X by 150-bp paired-end sequencing.

### PCR assay to detect residual plasmid

A pair of primers were designed in the outer and inner regions of the lentivirus packaging sequence to detect the residual plasmids and the integration of lentivirus (PF1: gtcggggctggcttaactat, PR1: taatcgccttgcagcacatc; LF1: tttccgggactttcgctttc, LR1: aagggacgtagcagaaggac). Then seven different templets were tested (H20; Blank genome DNA, 200 ng; pasmid, 10 ng; unconcentrated virus, 2 µl; concentrated virus, 2 µl; isolated genomic DNA, 1 day after lentivirus transduction, 200 ng; isolated genomic DNA, 5 day after lentivirus transduction, 200 ng). PCR conditions: 98 °C for 2 min, 25 cycles of 98 °C for 7 s, 65 °C for 15 s, and 72 °C for 15 s, and the final extension, 72 °C for 2 min. The PCR products were detected using a 2% agarose EGel EX (Life Technologies, Qiagen).

### Analysis of indel frequencies from deep-sequencing data

Illumina sequencing raw reads were processed using in-house Python scripts which combine a series of analysis tools. To avoid the influence of low-quality reads, FASTQ Masker (FASTX-Toolkit Version 0.0.13) was used to mask nucleotides with character “N” based on a quality score <10. Reads exactly matching scaffold sequence (gttttagagctagaaatagcaagttaaaataaggctagtccgttatcaacttgaaaaagtggcaccgagtcggtgcttttt) were extracted using AWK. Designed gRNA sequences were used as barcodes to demultiplexed the validated reads (reads contain less than 4 “N” nucleotides, barcode, and target region contains no “N”). Target regions were then extracted, and aligned to the index which was built from the designed gRNA sequences using Bowtie (version 1.2). For the plasmid library without genome editing, the target sequences not identical to the corresponding gRNA sequence were considered as synthesis/PCR errors. The target sequences in the edited library identical to the synthesis/PCR errors were removed from the edited library data. The remaining sequences not identical to the corresponding gRNA sequence were considered as edited sequences. Thus, the indel frequency of a gRNA was calculated by the following formula:

$${\mathrm{indel}}\,{\mathrm{frequency}} = \frac{{{\mathrm{Number}}\,{\mathrm{of}}\,{\mathrm{edited}}\,{\mathrm{reads}}\,{\mathrm{per}}\,{\mathrm{gRNA}}}}{{{\mathrm{Number}}\,{\mathrm{of}}\,{\mathrm{total}}\,{\mathrm{reads}}\,{\mathrm{per}}\,{\mathrm{gRNA}}}}$$

### Model summary of deep learning

The task of gRNA indel frequency prediction can be phrased as a regression problem. A mapping function was built to input the representations of gRNA sequence and outputs the indel frequency score in the range [0,1]. The mapping function here is Bidirectional long short-term memory neural network (BiLSTM) (a particular subclass of RNN, i.e., recurrent neural network). Unlike convolutional neural network (CNN) which treats DNA input as a grayscale image with only two possible values for each pixel rather than on real continuous-valued images in computer vision field, RNN is a special type of neural network designed for ordered sequence problems^55^. RNN is considered more natural to regard one DNA sequence as a sentence with four types of characters, namely A, C, G, and T, rather than an image, and thus related research work in natural language processing has offered valuable experience for DNA sequence modeling^56^. RNN have connections that have loops, adding feedback and memory to the networks over time or spatial sequences. This memory allows this type of network to learn and generalize across sequences of inputs rather than individual patterns. LSTM further takes the advantage of “gates” to control the degree of influence from the previous sequence features, which made it more flexible in controlling the memory. A BiLSTM exploits the order sensitivity of RNNs: it consists of using two LSTM layers, each of which processes the input sequence in one direction (chronologically and anti-chronologically), and then merging their representations. By processing a sequence both ways, a BiLSTM can catch patterns that may be overlooked by a unidirectional RNN. Borrowed from the natural language processing applications^57^, the gRNA sequence input was transformed to a matrix **x** = (***x***_1_, ***x***_2_, …, ***x***_*i*_, ***x***_*l*_), which was a ***l*** × 4 matrix (*l* = 21 means gRNA sequence length is 21 here, and ***x***_*i*_ means binary vector of four A, T, C, G nucleotides).

### Embedding

Embedding is quite useful for mapping a sparse matrix from input vector to a dense real-valued high-dimensional space, which can facilitate the training process. In natural language processing problems, typical model uses a word as its smallest input entity or a character level uses the character of alphabets as the smallest entity. Thus, we can consider the nucleotides in the gRNA sequence as word (naturally a character, too), and the gRNA sequence itself as a sentence. For this research, the input matrix ${\mathbf{x}} \in {\Bbb R}^{L \times 4}$ (*L* here is 21, the sequence length of gRNA) is projected to the dense real-valued space ${\mathbf{E}} \in {\Bbb R}^{L \times m}$ (**E** is embedding matrix, *m* is a hyperparameter corresponds to the embedding dimension) by the lookup matrix (embedding weight matrix) **W**_*m*_ (i.e., **E** = **x*W***_*m*_). Then, the embedding matrix **E** will be the input matrix of RNN.

### BiLSTM

As a de facto standard of RNN architectures, LSTM has achieved a very significant results in a variety of sequence-based tasks. However, the recently proposed Gated Recurrent Unit (GRU) architecture (a simplified LSTM architecture) mostly used in the context of machine translation^58^, did not show any significant improvement upon standard LSTM. The difference between the standard LSTM and simple RNN is the hidden layer. For example, given the input matrix ${\mathbf{E}} \in {\Bbb R}^{L \times m}$, a simple RNN produces matrix **H** of size *L* × *n* (where *n* is the RNN units). At each time step *l*, let ${\boldsymbol{e}}_l \in {\Bbb R}^m$ as the input column vector, ${\boldsymbol{h}}_{l - 1} \in {\Bbb R}^n$ as the previous hidden state vector, the current state ***h***_*l*_ by the following way:

1 $${\boldsymbol{h}}_l = \sigma \left( {{\boldsymbol{e}}_{\boldsymbol{l}}{\mathbf{W}} + {\boldsymbol{h}}_{l - 1}{\mathbf{U}} + {\boldsymbol{b}}} \right)$$

where **W**, **U**, and **b** are the trainable parameters, and **σ** is the nonlinear activation function. However, in LSTM, a well-designed gating mechanism avoids the “vanishing gradients” problem which made it more applicable on relatively long sequences. These gates can be trained to control the information flow of hidden neurons. The LSTM unit used in this research is implemented by replacing the aforementioned Eq. (1), which contains **e**_*l*_, **h**_*l*−1_, **c**_*l*−1_ as input, and produce **h**_*l*_, **c**_*l*_ (**c**_*l*_ is candidate state,):

2 $${\mathbf{i}}_l = {\mathrm{\sigma }}\left( {{\mathbf{e}}_l{\mathbf{W}}^{\mathrm{i}} + {\mathbf{h}}_{l - 1}{\mathbf{U}}^{\mathrm{i}} + {\mathbf{b}}^{\mathrm{i}}} \right)$$

3 $${\mathbf{f}}_l = {\mathrm{\sigma }}\left( {{\mathbf{e}}_l{\mathbf{W}}^{\mathrm{f}} + {\mathbf{h}}_{l - 1}{\mathbf{U}}^{\mathrm{f}} + {\mathbf{b}}^{\mathrm{f}}} \right)$$

4 $${\mathbf{c}}_l = {\mathbf{f}}_l \odot {\mathbf{c}}_{l - 1} + {\mathbf{i}}_l \odot {\mathrm{tanh}}\left( {{\mathbf{e}}_l{\mathbf{W}}^{\mathrm{c}} + {\mathbf{h}}_{l - 1}{\mathbf{U}}^{\mathrm{c}} + {\mathbf{b}}^{\mathrm{c}}} \right)$$

5 $${\mathbf{o}}_l = {\mathrm{\sigma }}\left( {{\mathbf{e}}_l{\mathbf{W}}^{\mathrm{o}} + {\mathbf{h}}_{l - 1}{\mathbf{U}}^{\mathrm{o}} + {\mathbf{b}}^{\mathrm{o}}} \right)$$

6 $${\mathbf{h}}_l = {\mathbf{o}}_l \odot {\mathrm{tanh}}(c_l)$$

where **W**, **U**, **V**, and **b** are the trainable parameters, σ(·),tanh(·), and ⊙ are element-wise sigmoid, hyperbolic tangent, and multiplication functions, respectively. **i**_*l*_, **f**_*l*_, and **o**_*l*_ are the input, forget, and output gates.

Due to the bidirectional reason, the model processes input data both in the forward and backward orders, allowing to combine the 5′ and 3′ gRNA sequence information in every time step. In parallel, one output is in forward order which is defined as ${{\mathop{\boldsymbol{h}}\limits^{\rightharpoonup} }}$, the other output is in reverse order which defined as $\overleftarrow {\boldsymbol{h}}$. Then, two hidden states are combined and generates an output vector **h**_*bi*_*l*_:

7 $${\mathbf{h}}_{{bi}\_{l}} = {\rm{{Bidirectional}}}\,({{\mathop{\mathbf{h}}\limits^{\rightharpoonup} }}_l,{{\mathop{\boldsymbol{h}}\limits^{\leftharpoonup} }}_l)$$

where Bidirectional(·) is a function used to combine the two output sequences. It can be a concatenating function, a summation function, an average function or a multiplication function. In this research, a concatenating function was adopted to get a **2n** output vector **h**_*bi*_*l*_ as the learned feature representation of gRNA sequence.

### Hand-crafted biological features

A lot of studies have shown that hand-crafted features may provide complementary information with CNN or RNN, which may give improved performance than only using the features automatically generated from the representation learning process. Motivated by this, we combined the secondary structural feature, GC content features, and thermodynamics features together with feature representations obtained from RNN to enhance the predictive capability of LSTM. Detailed hand-crafted features are shown in Supplementary Table 1. Here, we concatenate the biological feature vector ${\mathbf{h}}_{\mathbf{bio}} \in {\Bbb R}^p$, with **h**_*bi*_*l*_ from the previous BiLSTM stage and get **h**_new_ = concat ([**h**_bio_, **h**_*bi*_*l*_]) as the final representations of the gRNA sequence. The dimension of **h**_new_ is ***l*** × 2*n* + *p*.

### Fully connected layers

The previous concatenated feature representation **h**_new_ is the input of the fully connected layers. The number of layers, the hidden units, the activation function, and the dropout rate of each layer were determined by hyperparameter searching (described in the following Model training section). These layers are used to create final nonlinear combinations of features and for making predictions by the network.

### Model training

The number of validated deep-sequencing gRNAs is 55,604, 58,167, and 56,888 for eSpCas9(1.1) and SpCas9-HF1, respectively. Because of lacking appropriate third-party validation data sets and in order to get a more reliable training result, the data set were split into three subsets: 76.5% (42,537 for WT-SpCas9, 44,842 for eSpCas9(1.1), and 43,518 for SpCas9-HF1) for training, 8.5% (4726 for WT-SpCas9, 4982 for eSpCas9(1.1), and 4835 for SpCas9-HF1) for validation, and 15% (8341 for WT-SpCas9, 8793 for eSpCas9(1.1), and 8534 for SpCas9-HF1) for testing, respectively. The training data set was used to tune the parameters, the validation set was used to avoid over-fitting, and the testing set was used for the evaluation of the model performance. The performance metrics of the trained model are mean-squared error and Spearman correlation score. The hyperparameter searching space included 12 different hyperparameters, formed more than 237 billion parameter combinations. It is too large for a traditional grid search manner to get the optimized parameters. We, therefore, implemented a Bayesian optimization process to reduce the searching time, which has been confirmed to be more reliable than randomized grid search. The optimization program GpyOpt was developed by the machine-learning group of the University of Sheffield^59^. The number of initial random searching points was 30, which provided a clue for the best parameter, and then 300 acquisitions were implemented to attempt to get a global optimum within specified iterations. After getting the optimized hyperparameters of the models, a tenfold shuffled validation was implemented to evaluate the stability of the performance. The mean and standard deviation of the performance measures were obtained. The detailed hyperparameters are described in supplementary notes.

### Feature engineering and model comparisons

For the benchmark purpose, we trained simple Linear regression, L1-regularized linear regression (lasso regression), L2-regularized linear regression (Ridge regression), SVM regression, XGBoost regression, multilayer perceptron (MLP), CNN, and RNN models as baseline models. Like the deep-learning models, the hyperparameters were searched and determined by GpyOpt. The detailed hyperparameter configurations are shown in Supplementary Data 14–18.

Feature engineering was implemented by a recently developed method called Tree SHAP (SHapley Additive exPlanations), which combined SHAP value with XGBoost algorithm. Previously, feature selection for gRNA activity was usually implemented by tree ensemble methods^25,28^. However, the metrics of these methods (such as Gini importance or weight) lack of consistency (their value should not decrease when the true impact of that feature is increased) and accuracy (the sum of all the feature importance should sum up to the total importance)^49,60^. Tree SHAP addresses it by recent applications of game theory and develop fast exact tree solutions for SHAP values, which are the unique consistent and locally accurate additive feature attribution method based on expectations^60^. The final features adopted in the conventional algorithms were determined by the top 70% of the most important features which measured by SHAP value.

### Model interpretability

In addition to the prediction accuracy, we are also interested in the mechanisms of the deep-learning model. However, the multilayer nonlinear hidden units lead to a trade-off between the complexity and interpretability of deep neural network. The community has made a lot of efforts to understand the black box nature of the deep-learning model, which can be roughly divided into perturbation-based methods (suffers from the unacceptable computation cost) and backpropagation-based methods (may face problems of vanishing gradient and gradient discontinuity)^61^. A recently proposed method called DeepLift had proved especially useful in Recurrent Neural Networks where saturating activations like sigmoid or tanh are popular^62^, and was adopted by Lundberg and Lee to adapt DeepLIFT as a compositional approximation of SHAP values (introduced in the previous section, indicates the feature importance), leading to Deep SHAP. We use Deep SHAP to calculate the SHAP values to estimate the feature importance of sequence input into the best model.

### Tools used in the study

RNAfold 2.4.5^63^ was used to generate secondary structure features and thermodynamic features. Scikit-learn 0.19.1^64^ and Keras 2.1.6^65^ (https://github.com/fchollet/keras.) with TensorFlow 1.8.0^66^ as backend were used for conventional machine-learning algorithms and deep-learning models, respectively.

### Detection of indel frequency on endogenous target sites

To test the performance of seven algorithms for endogenous sites, we edited endogenous sites with randomly selected 100 gRNAs from the library (Supplementary Data 9). The epiCRISPR plasmid was modified to express gRNAs. Briefly, the EF1-Cas9 fragment (AgeI-NheI) on epiCRISPR was replaced by CMV promoter, resulting in epiCRISPR-hU6-gRNA vector; then, hU6 promoter from epiCRISPR-hU6-gRNA vecotor was replaced by mU6 promoter using Gibson Assembly (NEB), resulting in epiCRISPR-mU6-gRNA vector. The gRNA oligonucleotide pairs were annealed and cloned into BspQI sites of the epiCRISPR-mU6-gRNA vector. The plasmids were transfected into HEK293T cells expressing eSpCas9 or SpCas9-HF1, respectively. After 24 h of transfection, cells were selected with 2 µg/ml puromycin for the 4 days.

### Analysis of individual gRNA indel frequency

All PCR primers for T7EI and TIDE analysis are listed in Supplementary Data 20. Genomic DNA was extracted from cells at suitable time points after transfection or infection using QuickExtract DNA Extraction Solution (Epicentre) according to the manufacturer’s instructions. We amplified the target sequence by PCR with Q5 High-Fidelity 2X Master Mix (NEB) following the manufacturer’s instructions. PCR products were purified with Gel Extraction Kit (Qiagen) and cleavage by T7EI (NEB). Digested DNA was run on TBE gel. To calculate the indel efficiency, the intensity of band was analyzed using the ImageJ software. For TIDE, the purified PCR products were Sanger-sequenced, and each sequence was analyzed with the online TIDE software available at http://tide.nki.nl^67^.

### Detection of genotype and phenotype

We selected nine sgRNAs for three genes (SIRT1, SIRT2, and SIRT6) to examine the relationship between indel frequency and actual gene disruption. epiCRISPR-mU6-gRNA were transfected into 293T-SpCas9-HF1 cells, the gDNA and protein were extracted at 9 days after puromycin screening. The indel frequency detected by TIDE software, the gene disruption detected by western blot.

### Luciferase reporter assay

To establish luciferase reporter cell line, HEK293T cells in six-wells plates were transfected with 2 μg of mixed plasmids (pX458-AAVS1-gRNA and pAAVS1-luciferase-donnor) using Lipofectamine 2000 (Life Technologies) based on the manufacturer’s instruction. After 24 h, cells were selected with 10 μg/ml of blasticidin for 14 days. The cells expressing luciferase were plated in 48-wells plates and transfected with 0.2 μg plasmids expressing Cas9 nucleases and firefly gRNAs. After 5 days, cells were harvested, and luciferase activity was measured using Dual-Luciferase^®^ Reporter Assay System (Promega). Firefly luciferase activity was detected and normalized to renilla luciferase activity measured in the same samples. The levels of the normalized reporter luciferase activity were calculated relative to the levels in mock transfected cells expressing the same reporter.

### Statistical analysis

All the data are shown as the mean ± S.D. Statistical analyses were conducted using Microsoft Excel. Two-tailed, paired Student’s *t* tests were used to determine statistical significance when comparing two groups. A value of *p* < 0.05 was considered to be statistically significant.

### Reporting summary

Further information on research design is available in the Nature Research Reporting Summary linked to this article.

## Supplementary information

Supplementary Information

Supplementary Data 1

Supplementary Data 2

Supplementary Data 3

Supplementary Data 4

Supplementary Data 5

Supplementary Data 6

Supplementary Data 7

Supplementary Data 8

Supplementary Data 9

Supplementary Data 10

Supplementary Data 11

Supplementary Data 12

Supplementary Data 13

Supplementary Data 14

Supplementary Data 15

Supplementary Data 16

Supplementary Data 17

Supplementary Data 18

Supplementary Data 19

Supplementary Data 20

Reporting Summary

Description of Additional Supplementary Files

Source Data

## Data Availability

The full data sets of the indel rate can also be obtained in Supplementary Data 2. The raw sequencing data have been submitted to the NCBI Sequence Read Archive (SRA PRJNA522677 (https://www.ncbi.nlm.nih.gov/bioproject/522677)). The raw read counts can be obtained in Supplementary Data 19. The source data underlying Fig. 1b–g and Supplementary Figs. 1–5, 6c, 7b, 13, and 14 are provided as a Source Data file. The other data for this study are available from the corresponding author upon reasonable request.

## Code availability

All software codes of the study are available in GitHub (https://github.com/izhangcd/DeepHF). We built a website DeepHF for the access of all indel rate data (http://www.DeepHF.com/). One can input an Entrez Gene ID or official gene symbol to query the validated gRNA efficiencies for eSpCas9(1.1) and SpCas9-HF1.
