# Supplementary material for: Optimized CRISPR guide RNA design for two high-fidelity Cas9 variants by deep learning
Source: Nat Commun. 2019 Sep 19;10:4284. doi: 10.1038/s41467-019-12281-8 (PMC6753114; doi:10.1038/s41467-019-12281-8)
Supplement: Supplementary file 1 — Supplementary Information [file 41467_2019_12281_MOESM1_ESM.pdf]

Supplementary Information for

Optimized CRISPR guide RNA design for two high-fidelity Cas9 variants by deep learning

Daqi Wang[1+], Chengdong Zhang[1+] et al.

Supplementary Notes

Detailed hyperparameters searching space used in this study:

(1) XGBoost

I. Learning rate: (0.01, 0.12, 0.23, 0.34, 0.45, 0.56, 0.67, 0.78, 0.89, 1.0)

II. Sub sample: (0.5, 0.556, 0.611, 0.667, 0.722, 0.778, 0.833, 0.889, 0.944, 1.0)

III. Subsample ratio of columns when constructing each tree: (0.5, 0.556, 0. 611, 0.667, 0.722, 0.778, 0.833, 0.889, 0.944, 1.0)

IV. Maximum depth of a tree: (3, 4, 5, 6)

V. Number of boosted trees to fit: (100, 200, 300, 400, 500, 600, 700, 800, 900,1 000)

VI. L2 regularization term on weights: (1, 11, 21, …, 479, 489, 500)

VII. Objective: {'0':'reg: tweedie', '1':'reg: linear'}

(2) Multilayer perceptron

I. Number of fully connected layers: (1,2,3,4,5,6)

II. Number of unites in fully connected layers: (50, 60, 70,80, 90, 100, 120, 140, 160, 180, 200, 220, 240, 260, 280, 300, 320, 340, 360, 380, 400)

III. Fully connected layers dropout rate:(0.1,0.2,0.4,0.4,0.5,0.6,0.7)

IV. Batch size: (20,30, 40, 50,60, 70, 80,90, 100)

V. Epochs: (20, 25, 30, 35, 40, 45, 50, 55)

VI. Activation function: {'1':'relu', '2':'tanh', '3':'sigmoid', '4':'hard_sigmoid', '0':'elu'}

XII. Optimizer: {'1': SGD, '2': RMSprop, '3': Adagrad, '4': Adadelta, '5': Adam, '6': Adamax, '0': Nadam}

(3) CNN

Learning rate was fixed as 0.001.

I. Embedding dimensions: (16, 20, 24, 28, 32, 36, 40, 44, 48, 52, 56, 60, 64, 68)

II. Embedding dropout rate: (0.1,0.2,0.3)

III. Number of fully connected(FC) layers: (1,2,3,4,5,6)

IV. Number of unites in FC layers: (50, 60, 70,80, 90, 100, 120, 140, 160, 180, 200, 220, 240, 260, 280, 300, 320, 340, 360, 380, 400)

V. FC dropout rate:(0.1,0.2,0.4,0.4,0.5,0.6,0.7)

VI. Batch size: (20,30, 40, 50,60, 70, 80,90, 100)

VII. Epochs: (20, 25, 30, 35, 40, 45, 50, 55)

VIII. FC Activation function: {'1':'relu','2':'tanh', '3':'sigmoid', '4':'hard_sigmoid', '0':'elu'}

IX. Convolutional Layers:{

'0':

['1:2:3:4:5:6:7:8:9:10:11:12:13:14:15','20:20:20:20:20:20:20:20:20:20:20:20:20:20'],

'1':

['1:2:3:4:5:6:7:8:9:10:11:12:13:14:15','20:20:20:20:20:20:20:40:40:40:40:40:40:40'],

'2':

['1:2:3:4:5:6:7:8:9:10:11:12:13:14:15','20:20:20:20:40:40:40:40:80:80:80:80:80:80'],

'3':

['1:2:3:4:5:6:7:8:9:10:11:12:13:14:15','40:40:40:40:40:40:40:40:80:80:80:80:80:80'],

}

XII. Optimizer: {'1’: SGD, '2’: RMSprop, '3’: Adagrad, ‘4’: Adadelta, '5’: Adam, '6’: Adamax, '0’: Nadam}

Notion: In the hyperparameter of convolutional layers, the first element of the list is feature maps, the second element of the list is kernel sizes.

(4) RNN

Learning rate was fixed as 0.001.

X. Embedding dimensions: (16, 20, 24, 28, 32, 36, 40, 44, 48, 52, 56, 60, 64, 68)

XI. Embedding dropout rate: (0.1,0.2,0.3)

XII. RNN cell units: (20, 30, 40, 50, 60, 70, 80, 90, 100, 120, 140, 160, 180, 200, 220, 240)

XIII. RNN dropout rate: (0.1,0.2,0.4,0.4,0.5,0.6,0.7)

XIV. Recurrent RNN dropout rate: (0.1,0.2,0.4,0.4,0.5,0.6,0.7)

XV. Number of fully connected(FC) layers: (1,2,3,4,5,6)

XVI. Number of unites in FC layers: (50, 60, 70,80, 90, 100, 120, 140, 160, 180, 200, 220, 240, 260, 280, 300, 320, 340, 360, 380, 400)

XVII. FC dropout rate:(0.1,0.2,0.4,0.4,0.5,0.6,0.7)

XVIII. Batch size: (20,30, 40, 50,60, 70, 80,90, 100)

XIX. Epochs: (20, 25, 30, 35, 40, 45, 50, 55)

XX. FC Activation function: {'1':'relu','2':'tanh', '3':'sigmoid', '4':'hard_sigmoid', '0':'elu'}

XII. Optimizer: {'1’: SGD, '2’: RMSprop, '3’: Adagrad, ‘4’: Adadelta, '5’: Adam, '6’: Adamax, '0’: Nadam}

The feature engineering was implemented by SHAP and XGBoost algorithm, the hyper parameters of XGBoost were shown in **Supplemental Data 4**. The feature importance was measured by SHAP value. After excluding 0-valued features, top 70% of the remaining most important features were selected as the final features used in the other conventional models. The corresponding threshold is 1.4434 for eSpCas9 and 1.3006 for Cas9- HF1 respectively. There left 696 and 701 features to be included in the other baseline models, respectively. We found that stem-loop, melting temperature, and free energy were the top important features in both eSpCas9 and Cas9- HF1. These complicated features can hardly get from the original sequence. This further demonstrates the need for biological features (especially secondary structure and thermodynamics) as complementary information in the deep learning models.

For RNN+biofeature models, the hyperparameters of the two different highly specific Cas9s almost converge to the same optimized combinations. Except for embedding dimensions (eSpCas9,44; Cas9-HF1, 48), number of fully connected layers

(eSpCas9,3; Cas9-HF1,2) and dropout rate of fully connected layers (eSpCas9,0.4; Cas9-HF1,0.5). In fact, neural network is quite a flexible model which can tolerance the changes of parameters in a limited range. The optimal parameter configurations for the other nine hyperparameters are all the same. The detailed hyperparameters for DeepHF and other models were showed in Supplemental Data 14-18.

Supplementary Figures

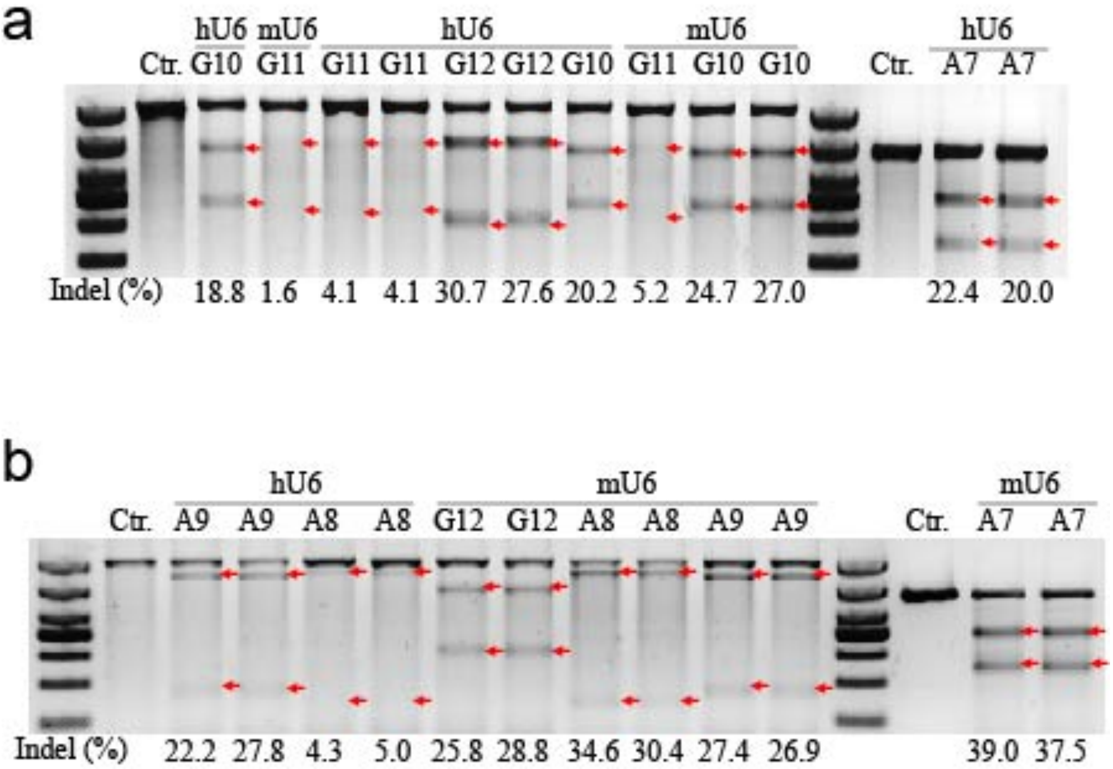

Supplementary Figure 1. Examples of T7EI digestion for hU6 promoter and mU6 promoter at day 3 post-transfection in HEK293T cells. a gRNAs initiated with G. **b** gRNAs initiated with A but free of G at 1-4 nucleotides. Red arrows indicate the expected bands after digestion; Ctr: cells without plasmid transfection. Source data are provided as a Source Data file.

a

A10: AGGGCAGGAAGAGGACACAG
A11: AAGCAGCACTCTGCCCTCGT
A12: ATGGGAGCAGCTGGTCAGAG
A13: ACTGAGGCTACATAGGGTTA
A14: ATGGGTCTAACATTCACAGA
A15: AGGTGTGGTTCCAGAACCGG
A16: AGGCCCCAGTGGCTGCTCTG
A17: ATTGCCACGAAGCAGGCCAA
A18: ACCGGAGGACAAAGTACAAA

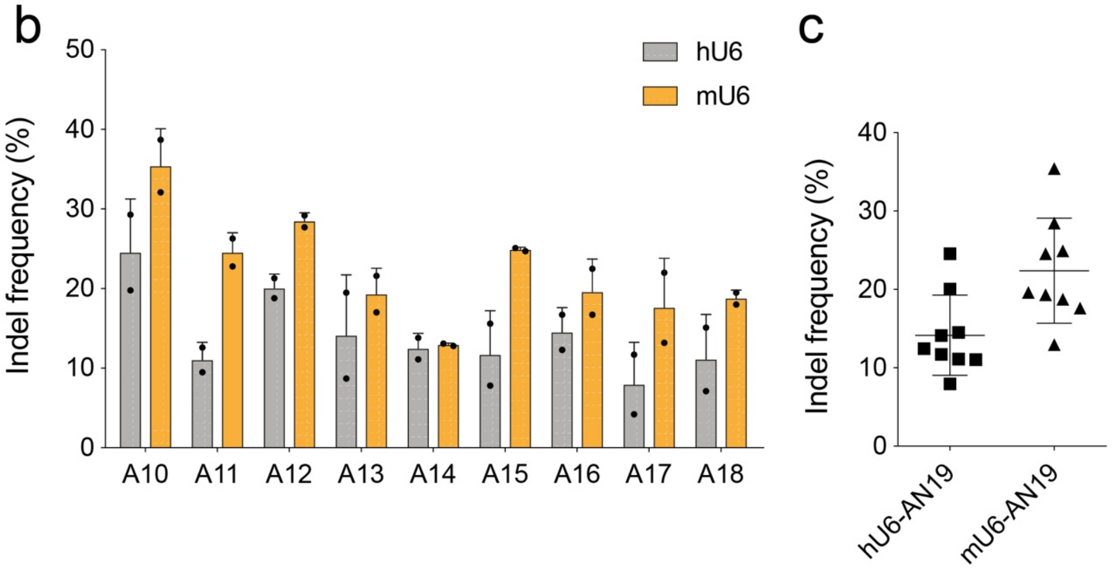

Supplementary Figure 2. Both hU6 promoter and mU6 promoter enabled to transcript gRNAs initiated with A for genome editing. a Nine gRNA sequences initiated with A but contained G at 1-4 nucleotides. **b** Indel frequencies induced by hU6 promoter and mU6 promoter. Data are shown as mean ± s.d. (n = 2) **c** Summary of the indel frequencies. Source data are provided as a Source Data file.

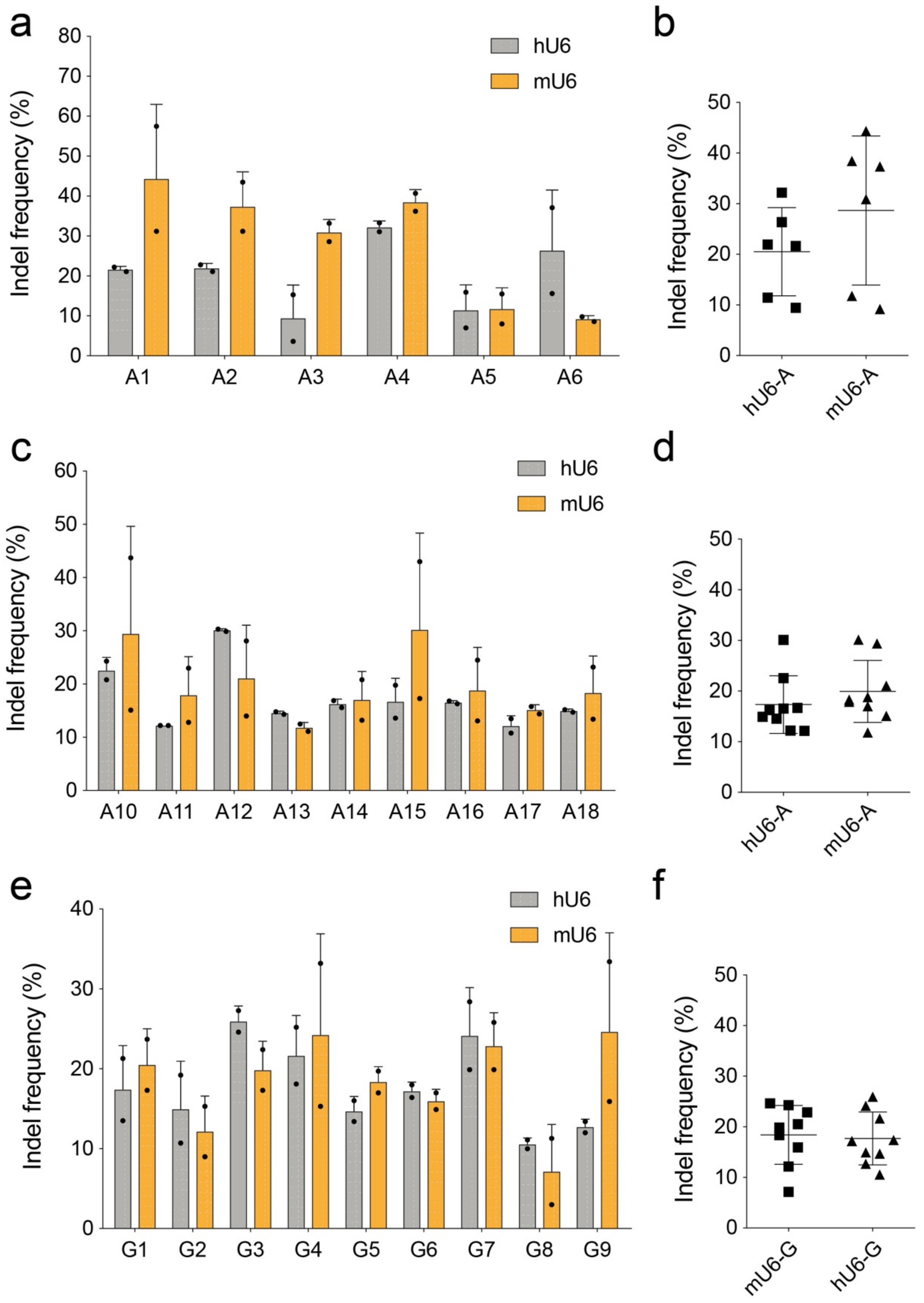

Supplementary Figure 3. Comparison of hU6 promoter and mU6 promoter activity in HeLa cells. a, **b** Indel frequencies for six gRNAs initiated with A but free of G at 1-4 nucleotides. **c**, **d** Indel frequencies for nine gRNAs initiated with A but contained G at 1-4 nucleotides. **e**, **f** Indel frequencies for nine gRNAs initiated with G. Data are shown as mean ± s.d. (n = 2). gRNA sequences were shown in **Table 1** and **Supplementary Fig. 2a**. Source data are provided as a Source Data file.

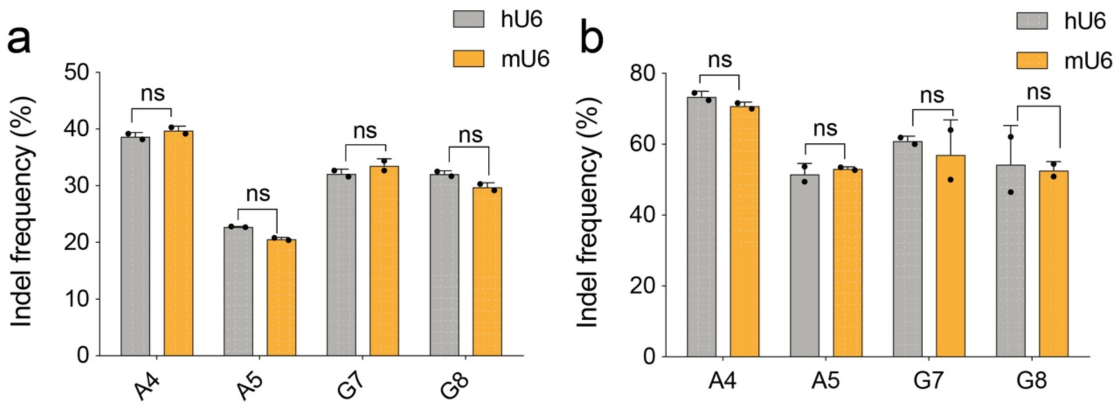

Supplementary Figure 4. hU6 and mU6 promoters promoted genome editing in a lentivirus vector. a The indel frequencies were detected at day 3. **b** The indel frequencies were detected at day 5. Data are shown as mean ± s.d. P>0.05; P<0.05 by 2way ANOVA (n = 2 per group). n.s. = not significant. gRNA sequences were shown in **Table 1.** Source data are provided as a Source Data file.

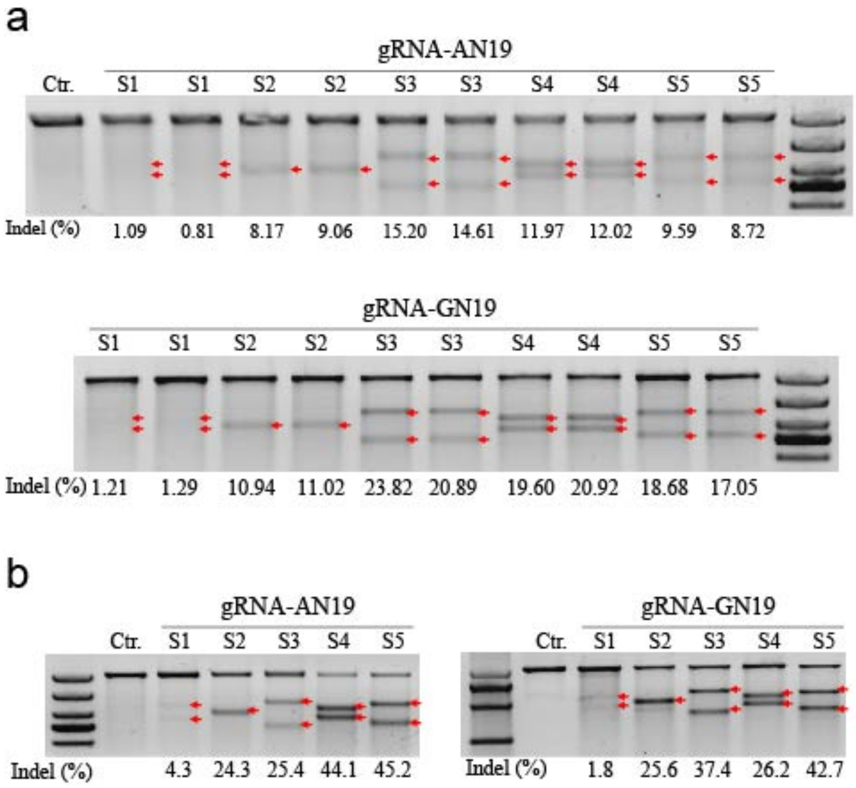

Supplementary Figure 5. Gel pictures of T7EI digestion with gRNAs initiated with A or G driven by mouse U6 promoter. a The gRNA activity was analyzed at day 3 post-transfection. **b** The gRNA activity was analyzed at day 5 post-transfection. Red arrows indicate the expected bands after digestion; Ctr: cells without plasmid transfection. Source data are provided as a Source Data file.

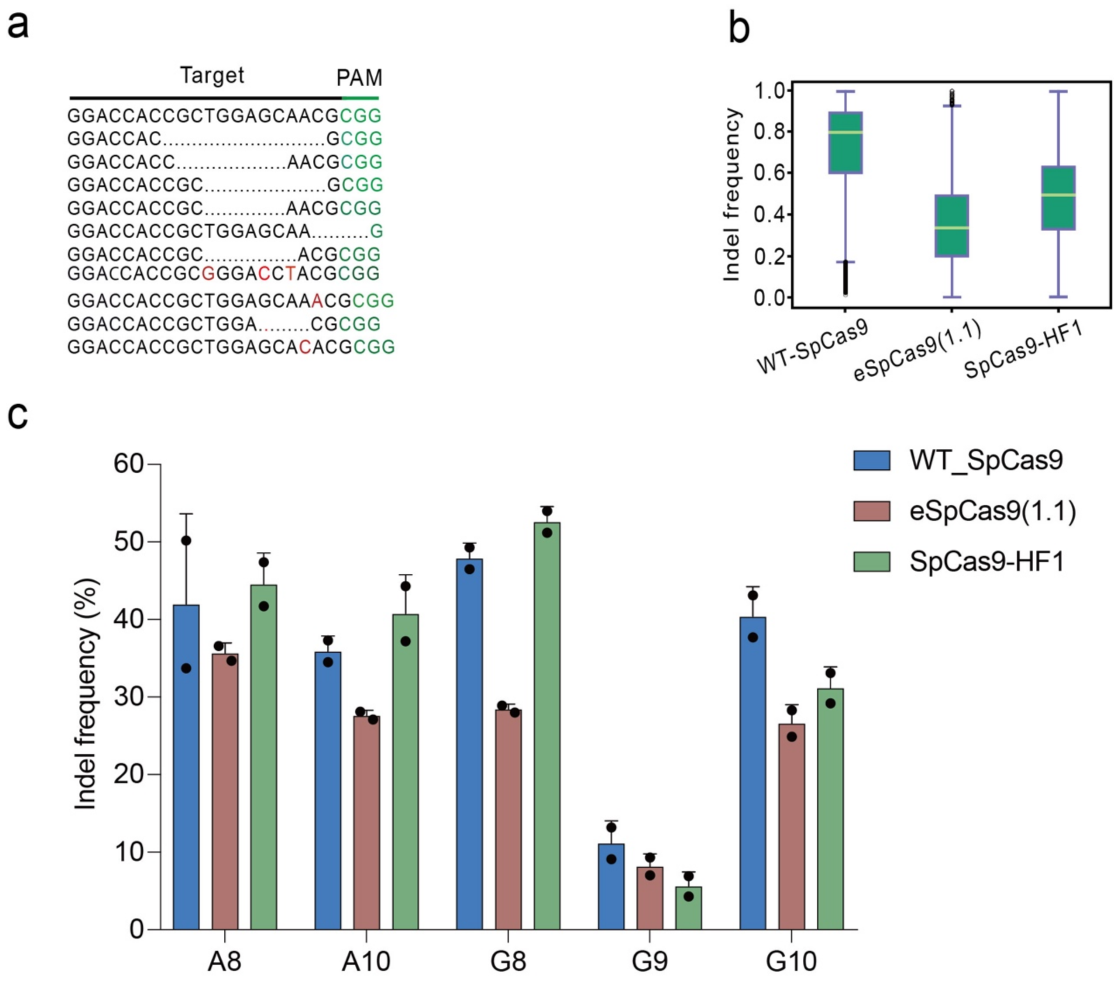

Supplementary Figure 6. Comparison of three Cas9 activity. a An example of indels generated at integration targets of EIF2B2 gene detected by deep sequencing. PAM sequences were shown in green. The insertions and mismatches were shown in red. **b** The boxplot results of indel frequency for high-throughput test of gRNA activity. **c** Indel frequency was detected after transient transfection of Cas9 and gRNA in HEK293T cells (n = 2 per group). Data are shown as mean ± s.d. gRNA sequences were shown in **Table 1**. Source data are provided as a Source Data file.

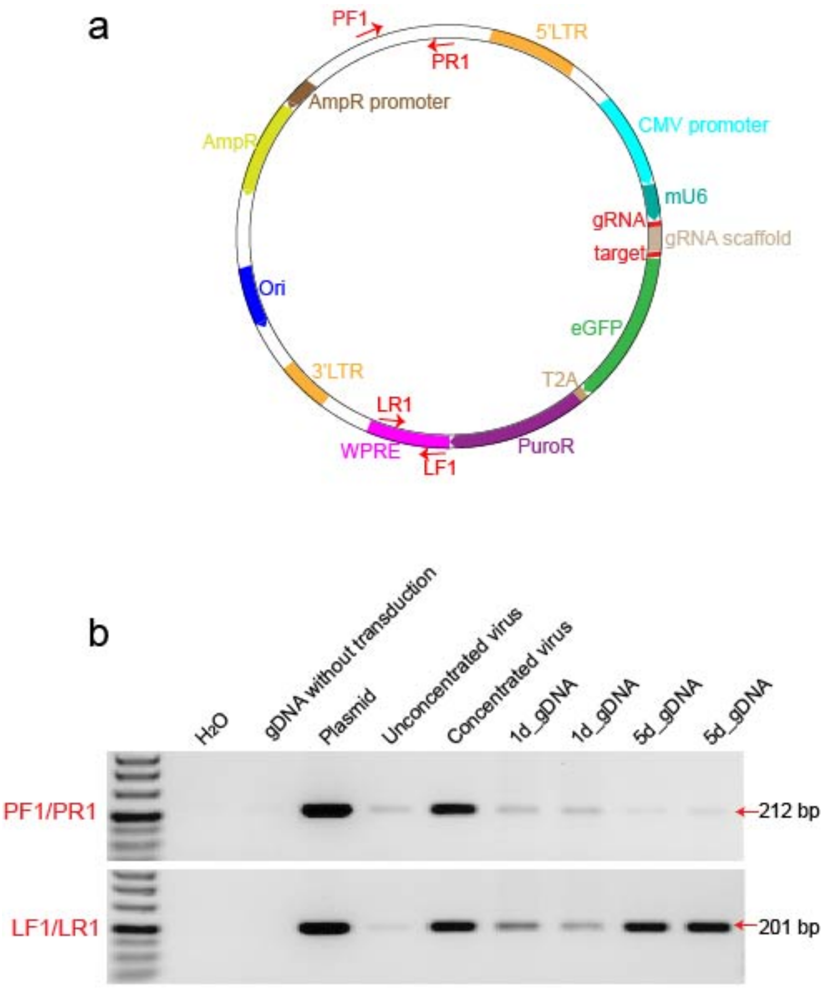

Supplementary Figure 7. Residual plasmid DNA detected in the transduced cells. **a** Primer design. **b** Minimal residual plasmid DNA could be detected with primers PF1/PR1 in cells five days after transduction. The integrated lentivirus DNA could be detected with primers LF1/LR1. The genomic DNA was prepared in two independent transduction assay. 1d_gDNA, genomic DNA prepared one day after transduction; 5d_gDNA, genomic DNA prepared five days after transduction. Source data are provided as a Source Data file.

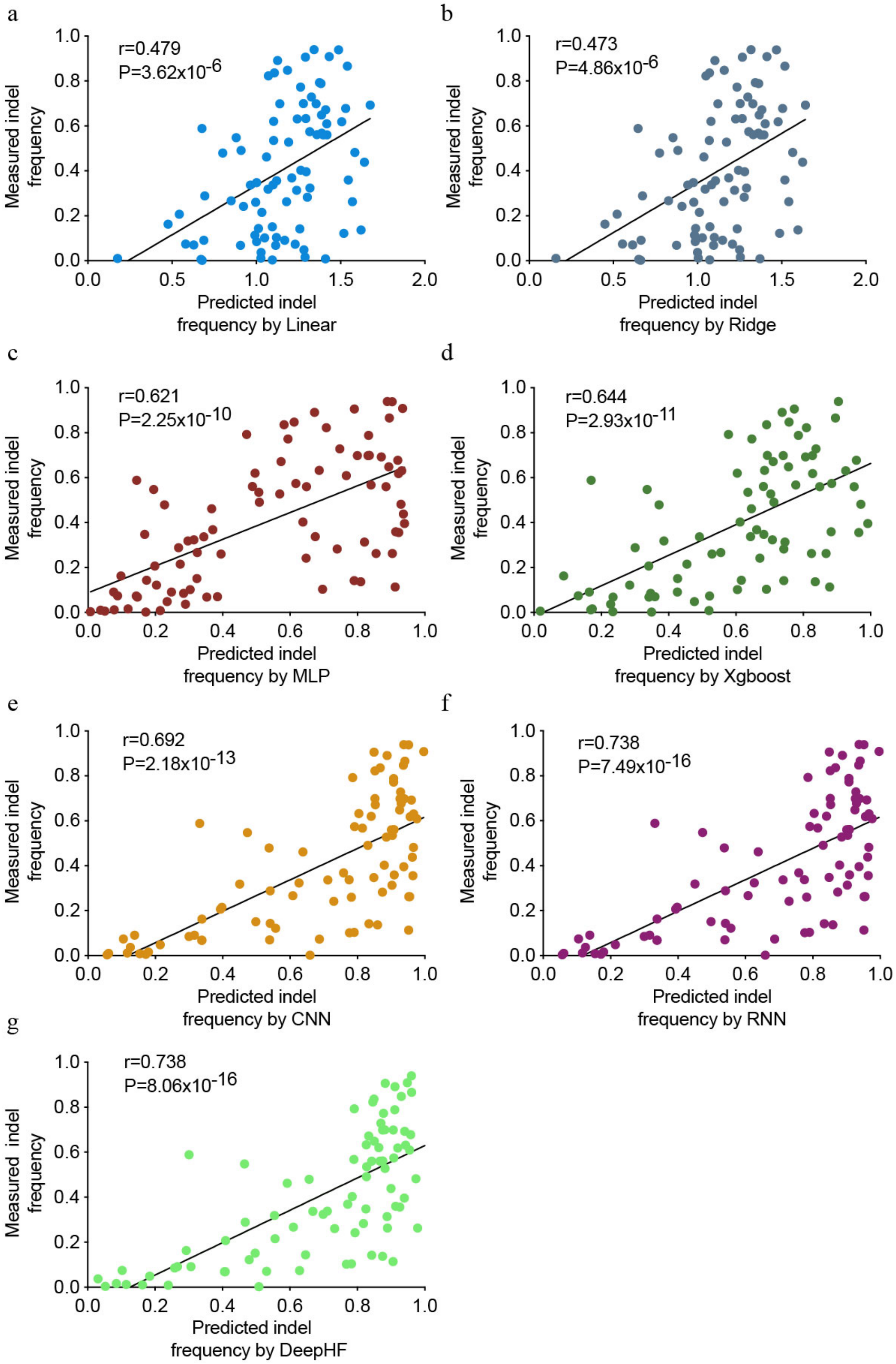

Supplementary Figure 8. The Spearman correlation between endogenous indel frequency and prediction score for WT-SpCas9. a-g The Spearman correlation between Linear, Ridge, MLP, XGBoost, CNN, RNN, DeppHF and endogenous loci, respectively.

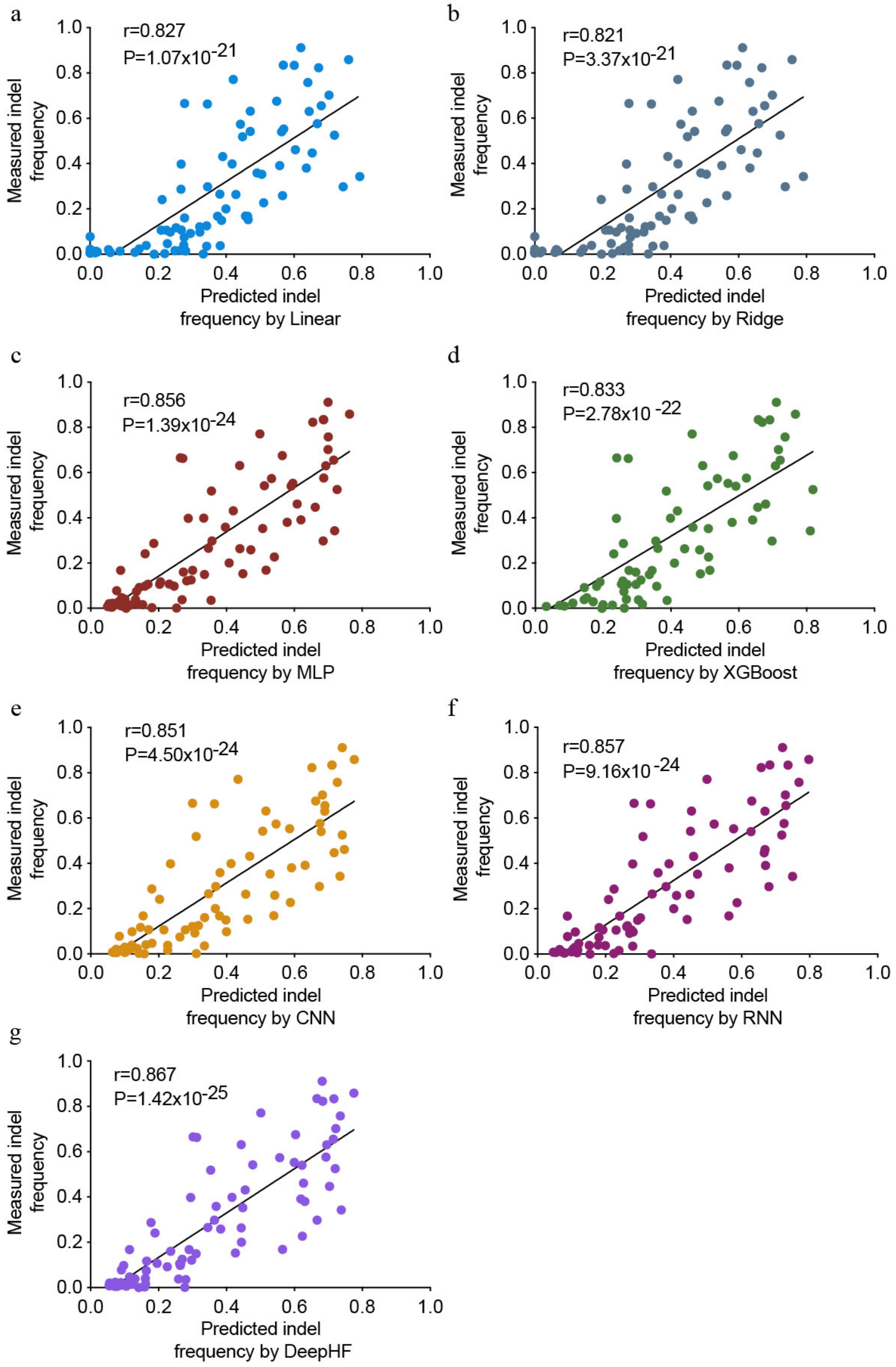

Supplementary Figure 9. The Spearman correlation between endogenous indel frequency and prediction score for eSpCas9. a-g The Spearman correlation between Linear, Ridge, MLP, XGBoost, CNN, RNN, DeppHF and endogenous loci, respectively.

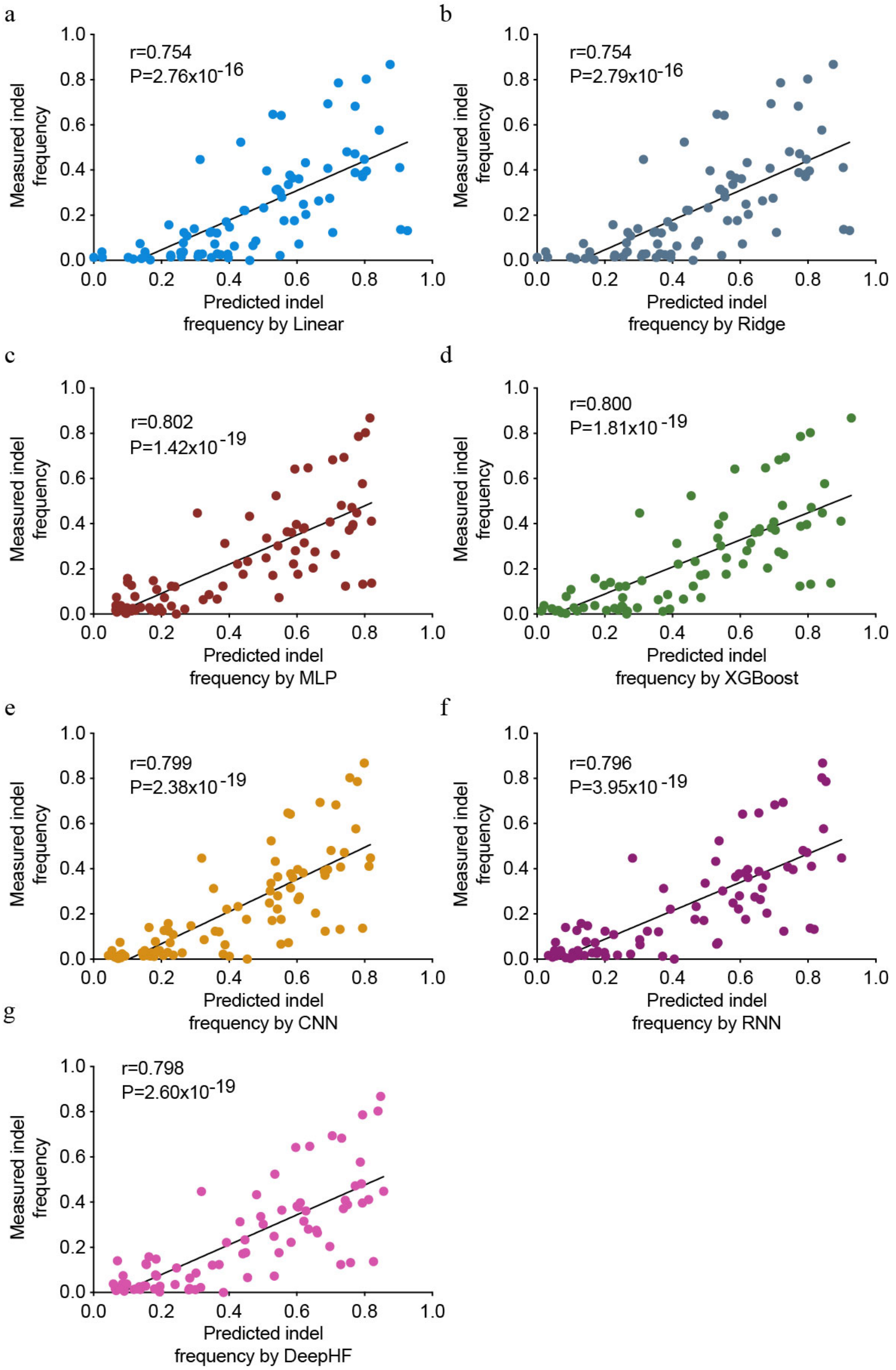

Supplementary Figure 10. The Spearman correlation between endogenous indel frequency and prediction score for SpCas9-HF1. a-g The Spearman correlation between Linear, Ridge, MLP, XGBoost, CNN, RNN, DeppHF and endogenous loci, respectively.

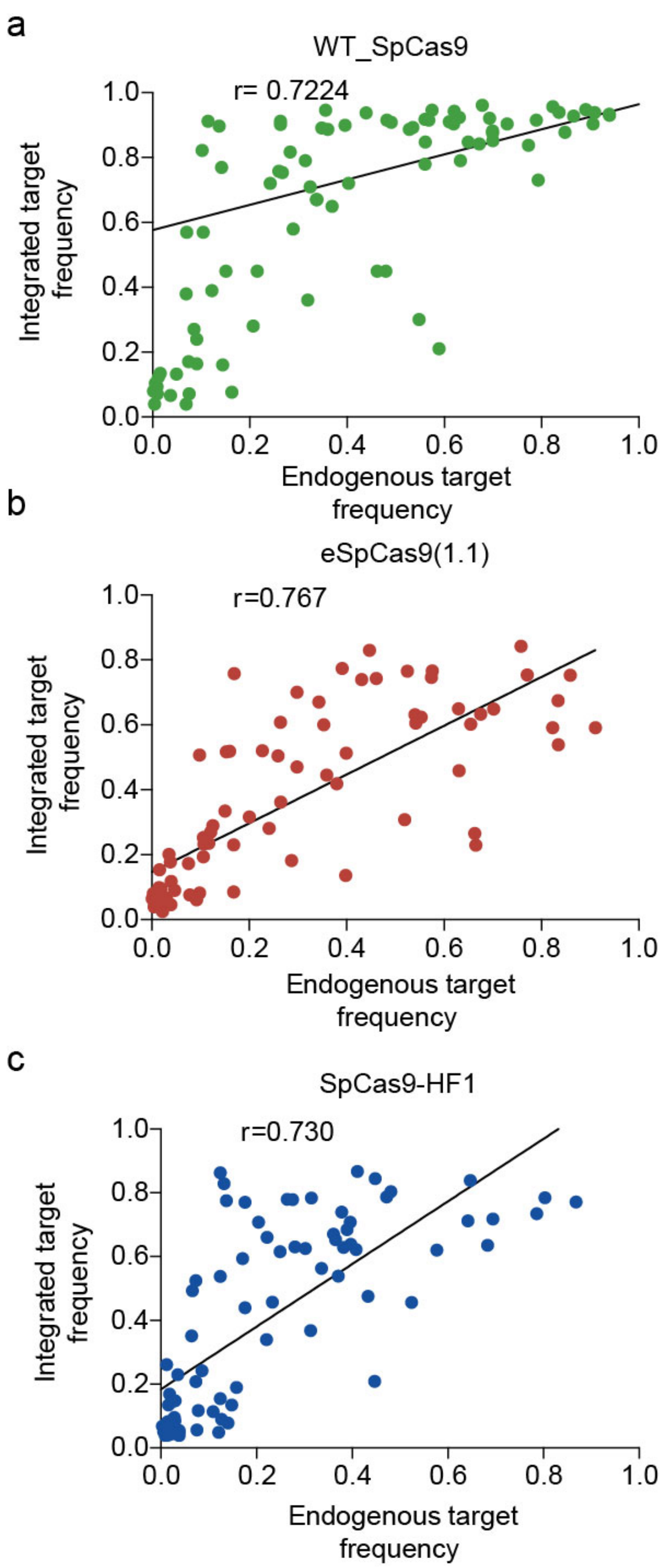

Supplementary Figure 11. The Spearman correlation of indel frequency between integrated targets and endogenous targets. a-c The Spearman correlation for WT-SpCas9, eSpCas9)1.1) and SpCas9-HF1, respectively.

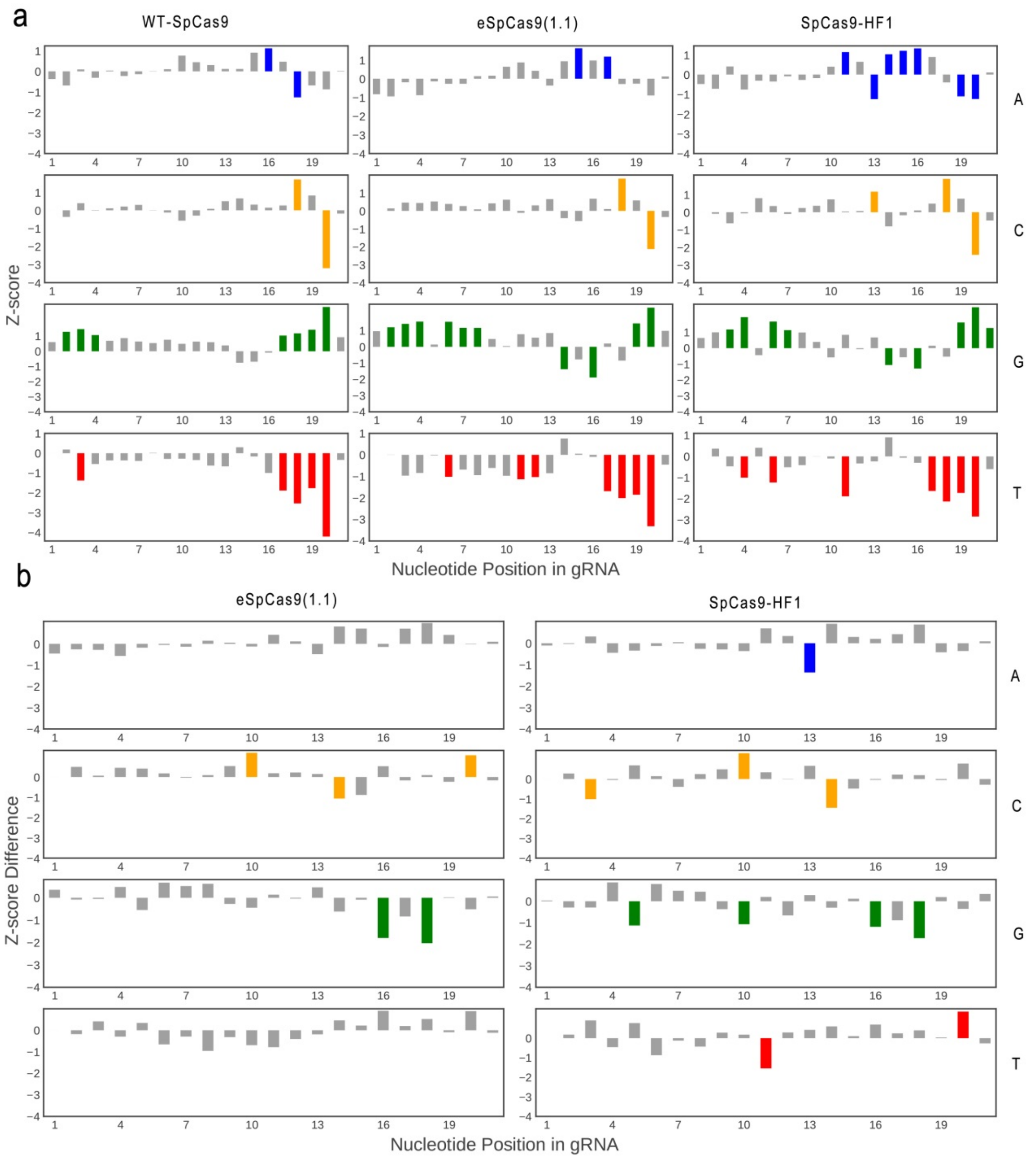

Supplementary Figure 12. Nucleotide contributions to gRNA activity revealed by Deep SHAP. a Deep SHAP values were rescaled by the Z-score (i.e. standardization). The height of the bar represented the Z-score. Colored bars represented Z-score above 1 or below -1. The left, middle, and right columns represent Wt-SpCas9, eSpCas9(1.1), and HF1-SpCas9, respectively. **b** The difference of Z-score between eSpCas9(1.1)/HF1-SpCas9 and WT-SpCas9. The height of the bar represented the differnce of Z-score. Colored bars represented difference of Z-score above 1 or below -1.

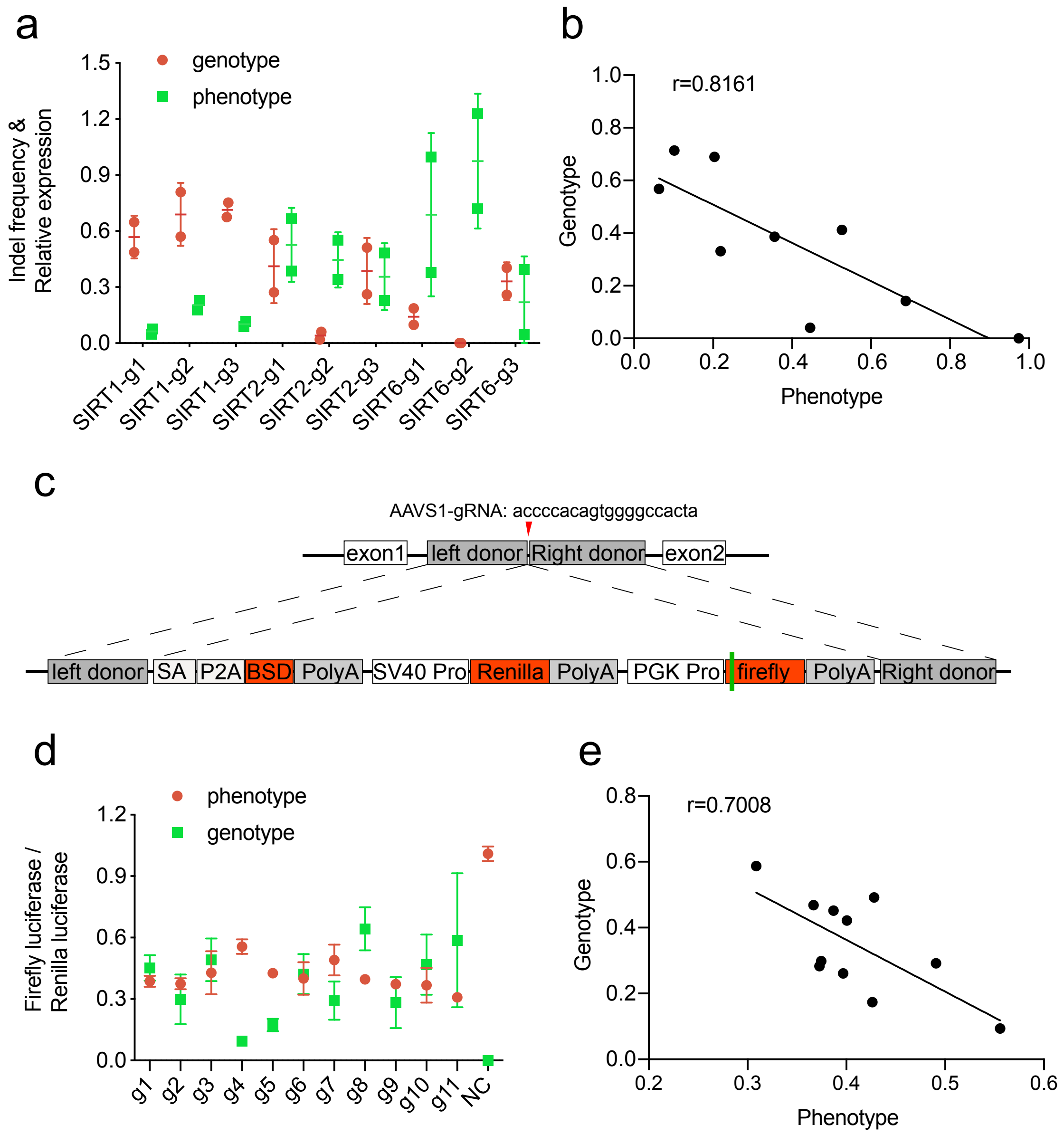

Supplementary Figure 13. The relationship between indel frequency and actual gene disruption. a After nine days of genome editing, indel frequencies(genotype) were measured by TIDE; protein expression(phenotype) was measured with Western blot. Data are shown as mean ± s.d. (n = 2 per group). **b** The correlation between indel frequency and protein expression was calculated by Pearson Correlation. **c** Schematic diagram of experimental design. Renilla and firefly were integrated into AAVS1 site in HEK293T cells by using genome editing technology. Red triangle shows the targeted site of CRISPR/Cas9; green bar on firefly gene shows the targeted site of gRNA. **d** After five days of genome editing, indel frequencies were measured by TIDE; luciferase activity was measured using Dual-Luciferase® Reporter Assay System. Data are shown as mean ± s.d. (n = 2 per group). **e** The correlation between genotype and phenotype was tested by Pearson Correlation analysis. Source data are provided as a Source Data file.

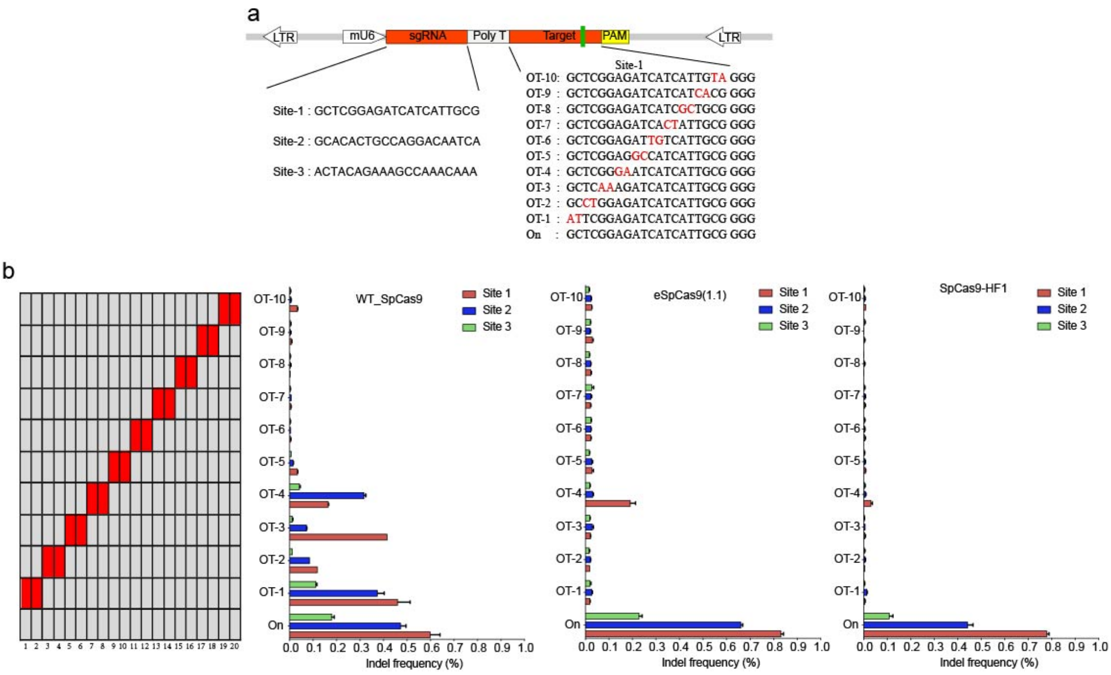

Supplementary Figure 14. Compare specificity of gRNAs with different activity. **a** Schematic diagram of the guide-target pair strategy. Three sgRNA sequences as well as target sequences for sgRNA-1 are shown below. Two base pairs of mismatch were indicated in red. Green box indicated mismatch. **b** Off-target mutations at each sites were detected by deep sequencing for three Cas9 nucleases. Schematic diagram of the mismatches were shown on the left. Data were shown as mean ± s.d. (n = 2 per group). Source data are provided as a Source Data file.

acgcgtgtagtcttatgcaatactcttgtagtcttgcaacatggtaacgatgagttagcaacatgccttacaaggagagaaaa
agcaccgtgcatgccgattggtggaagtaaggtggtacgatcgtgccttattaggaaggcaacagacgggtctgacatgg
attggacgaaccactgaattgccgcattgcagagatattgtatttaagtgcctagctcgatacaataaacgggtctctctggtt
agaccagatctgagcctgggagctctctggctaactagggaacccactgcttaagcctcaataaagcttgccttgagtgctt
caagtagtgtgtgcccgtctgttgtgtgactctggtaactagagatccctcagacccttttagtcagtgtggaaaatctctagca
gtggcgcccgaacagggacctgaaagcgaaagggaaaccagagctctctcgacgcaggactcggcttgctgaagcgc
gcacggcaagaggcgaggggcggcgactggtgagtacgccaaaaattttgactagcggaggctagaaggagagagat
gggtgcgagagcgtcagtattaagcgggggagaattagatcgcgatgggaaaaaattcggttaaggccagggggaaag
aaaaaatataaattaaaacatatagtatgggcaagcagggagctagaacgattcgcagttaatcctggcctgttagaaacat
cagaaggctgtagacaaatactgggacagctacaaccatcccttcagacaggatcagaagaacttagatcattatataatac
agtagcaaccctctattgtgtgcatcaaaggatagagataaaagacaccaaggaagctttagacaagatagaggaagagc
aaaacaaaagtaagaccaccgcacagcaagcggccactgatcttcagacctggaggaggagatatgagggacaattgg
agaagtgaattatataaatataaagtagtaaaaattgaaccattaggagtagcacccaccaaggcaaagagaagagtggtg
cagagagaaaaaagagcagtgggaataggagctttgttccttgggttcttgggagcagcaggaagcactatgggcgcag
cctcaatgacgctgacggtacaggccagacaattattgtctggtatagtgcagcagcagaacaatttgctgagggctattga

ggcgcaacagcatctgttgcaactcacagtctggggcatcaagcagctccaggcaagaatcctggctgtggaaagatacc
taaaggatcaacagctcctggggatttggggttgctctggaaaactcatttgcaccactgctgtgccttggaatgctagttgg
agtaataaatctctggaacagattggaatcacacgacctggatggagtgggacagagaaattaacaattacacaagcttaat
acactccttaattgaagaatcgcaaaaccagcaagaaaagaatgaacaagaattattggaattagataaatgggcaagtttgt
ggaattggtttaacataacaaattggctgtggtatataaaattattcataatgatagtaggaggcttggtaggtttaagaatagtt
tttgctgtactttctatagtgaatagagttaggcagggatattcaccattatcgtttcagacccacctcccaaccccgaggga
cccgacaggcccgaaggaatagaagaagaaggtggagagagagacagagacagatccattcgattagtgaacggatct
cgacggttaacttttaaaagaaaaggggggattggggggtacagtgcaggggaaagaatagtagacataatagcaacag
acatacaaactaaagaattacaaaaacaaattacaaaaattcaaaattttatcgatactagtaaggatctgcgatcgcgacatt
gattattgactagttattaatagtaatcaattacggggtcattagttcatagcccatatatggagttccgcgttacataacttacgg
taaatggcccgcctggctgaccgcccaacgacccccgcccattgacgtcaataatgacgtatgttcccatagtaacgccaa
tagggactttccattgacgtcaatgggtggagtatttacggtaaactgcccacttggcagtacatcaagtgtatcatatgccaa
gtacgccccctattgacgtcaatgacggtaaatggcccgcctggcattatgcccagtacatgaccttatgggactttcctactt
ggcagtacatctacgtattagtcatcgctattaccatggtgatgcggttttggcagtacatcaatgggcgtggatagcggtttg
actcacggggatttccaagtctccaccccattgacgtcaatgggagtttgttttggcaccaaaatcaacgggactttccaaaat
gtcgtaacaactccgccccattgacgcaaatgggcggtaggcgtgtacggtgggaggtctatataagcagtctagagatcc
gacgccgccatctctaggcccgcgccggcccctcgcacagacttgtgggagaagctcggctactcccctgccccggtta
atttgcatataatatttcctagtaactatagaggcttaatgtgcgataaaagacagataatctgttctttttaatactagctacatttt
acatgataggcttggatttctataagagatacaaatactaaattattattttaaaaaacagcacaaaaggaaactcaccctaact
gtaaagtaattgtgtgttttgagactataaatatgcatgcgagaaaagccttgtttaaac(PmeI)ttcgaatgcttttgtagctg
ttgtgcatgcacctagaggaaaacctacaacatttgttattcctacatttgtgccttttaataattctttacaatagcttgttcaatat
gaattaacacaaactgttgcaaaatcaaattcaattgcttcatcacataattgtttaatttcagctttcgtagcatcttgttttaataa
tgtgtgatctatatatttgtttagtttcatttttctcctatatattcatttttaattttaattctttaataatttcgtctactttaactttagcgt
tttgaacagattcaccaacacctataaaataaatttttagtttaggttcagttccacttgggcgaacagcaaatcatgacttatctt
ctaaataaaattttagtaagtcttgtcctggcatattatacattccatcgatgtagtcttcaacattaacaactttaagtccagcaat
ttgagttaagggtgttgctctcaatgatttcattaatggttcaatttttaatttcttttcttctggtttaaaattcaagtttaaagtgaaa
gtgtaatatgcacccatttctttaaataaatcttctaaatagtctactaatgttttattttgtttttataaaatcaagcagcctctgcta
ttaatatagaagcttgtattccatctttatctctagctgagtcatcaattacatatccataactttcttcataagcaaaaacaaaattt
aatccgttatcttcttctttagcaatttctctacccattcatttaaatccagttaaagtttttacaatattaactccatatttttcatgagc
gattctatcacccaaatcacttgttacaaaacttgaatatagagccggatttttttggaatgctatttaagcgttttagatttgataat
tttcaatcaattaaaattggtcctgtttgatttccatctaatcttacaaaatgaccatcatgttttattgccattccaaatctgtcagc
atctgggtcattcataataataatatctgcatcatgtttaataccatattcaagcggtatttttcatgcaggatcaaattctggattt
ggatttacaacatttttaaatgtttcatcttcaaatgcatgctcttcaacctcaataacgttatatcctgattcacgtaatatttttggg
gtaaatttagttcctgttccattaactgcgctaaaaataatttttaaatctttttttagcttcttgctctttttttgtacgtctctgttttagag
ctagaaatagcaagttaaaataaggctagtccgttatcaacttgaaaaagtggcaccgagtcggtgctttttttgaattctagatc
ttgagacaaatggcagtattcatccacaattttaaaagaaaaggggggattggggggtacagtgcaggggaaagaatagta
gacataatagcaacagacatacaaactaaagaattacaaaaacaaattacaaaaattcaaaattttcgggtttattacaggga
cagcagagatccactttggcgccggctcgagcacgtg(PmlI)agcaagggcgaggagctgttcaccggggtggtgcc
catcctggtcgagctggacggcgacgtaaacggccacaagttcagcgtgtccggcgagggcgagggcgatgccaccta
cggcaagctgaccctgaagttcatctgcaccaccggcaagctgcccgtgccctggcccaccctcgtgaccaccctgacct
acggcgtgcagtgcttcagccgctaccccgaccacatgaagcagcacgacttcttcaagtccgccatgcccgaaggctac
gtccaggagcgcaccatcttcttcaaggacgacggcaactacaagacccgcgccgaggtgaagttcgagggcgacacc
ctggtgaaccgcatcgagctgaagggcatcgacttcaaggaggacggcaacatcctggggcacaagctggagtacaact
acaacagccacaacgtctatatcatggccgacaagcagaagaacggcatcaaggtgaacttcaagatccgccacaacatc

gaggacggcagcgtgcagctcgccgaccactaccagcagaacacccccatcggcgacggccccgtgctgctgcccga
caaccactacctgagcacccagtccgccctgagcaaagaccccaacgagaagcgcgatcacatggtcctgctggagttc
gtgaccgccgccgggatcactctcggcatggacgagctgtacaagggatccgcggccgctgagggcagaggaagtctt
ctaacatgcggtgacgtggaggagaatcccggccct tccgggatgaccgagtacaagcccacggtgcgcctcgccaccc
gcgacgacgtccccagggccgtacgcaccctcgccgccgcgttcgccgactaccccgccacgcgccacaccgtcgatc
cggaccgccacatcgagcgggtcaccgagctgcaagaactcttcctcacgcgcgtcgggctcgacatcggcaaggtgtg
ggtcgcggacgacggcgccgcggtggcggtctggaccacgccggagagcgtcgaagcgggggcggtgttcgccgag
atcggcccgcgcatggccgagttgagcggttcccggctggccgcgcagcaacagatggaaggcctcctggcgccgcac
cggcccaaggagcccgcgtggttcctggccaccgtcggcgtctcgcccgaccaccagggcaagggtctgggcagcgc
cgtcgtgctccccggagtggaggcggccgagcgcgccggggtgcccgccttcctggagacctccgcgccccgcaacct
cccettctacgagcggctcggcttcaccgtcaccgccgacgtcgaggtgcccgaaggaccgcgcacctggtgcatgacc
cgcaagcccggtgcctgaatctaggtcgacaatcaacctctggattacaaaatttgtgaaagattgactggtattcttaactat
gttgctccttttacgctatgtggatacgctgctttaatgcctttgtatcatgctattgcttcccgtatggctttcattttctcctccttgt
ataaatcctggttgctgtctctttatgaggagttgtggcccgttgtcaggcaacgtggcgtggtgtgcactgtgtttgctgacg
caacccccactggttggggcattgccaccacctgtcagctcctttccgggactttcgctttccccctccctattgccacggcg
gaactcatcgccgcctgccttgcccgctgctggacaggggctcggctgttgggcactgacaattccgtggtgttgtcgggg
aaatcatcgtcctttccttggctgctcgcctgtgttgccacctggattctgcgcgggacgtccttctgctacgtcccttcggccc
tcaatccagcggaccttccttcccgcggcctgctgccggctctgcggcctcttccgcgtcttcgccttcgccctcagacgag
tcggatctccctttgggccgcctccccgcctggtacctttaagaccaatgacttacaaggcagctgtagatcttagccactttt
aaaagaaaaggggggactggaagggctaattcactcccaacgaaaataagatctgctttttgcttgtactgggtctctctggt
tagaccagatctgagcctgggagctctctggctaactagggaacccactgcttaagcctcaataaagcttgccttgagtgctt
caagtagtgtgtgcccgtctgttgtgtgactctggtaactagagatccctcagacccttttagtcagtgtggaaaatctctagca
gtagtagttcatgtcatcttattattcagtatttataacttgcaaagaaatgaatatcagagagtgagaggaacttgtttattgcag
cttataatggttacaaataaagcaatagcatcacaaatttcacaaataaagcatttttttcactgcattctagttgtggtttgtccaa
actcatcaatgtatcttatcatgtctggctctagctatcccgcccctaactccgcccagttccgcccattctccgccccatggct
gactaattttttttatttatgcagaggccgaggccgcctcggcctctgagctattccagaagtagtgaggaggcttttttggag
gcctagacttttgcagagacggcccaaattcgtaatcatggtcatagctgtttcctgtgtgaaattgttatccgctcacaattcc
acacaacatacgagccggaagcataaagtgtaaagcctggggtgcctaatgagtgagctaactcacattaattgcgttgcg
ctcactgcccgctttccagtcgggaaacctgtcgtgccagctgcattaatgaatcggccaacgcgcggggagaggcggttt
gcgtattgggcgctcttccgcttcctcgctcactgactcgctgcgctcggtcgttcggctgcggcgagcggtatcagctcact
caaaggcggtaatacggttatccacagaatcaggggataacgcaggaaagaacatgtgagcaaaaggccagcaaaagg
ccaggaaccgtaaaaaggccgcgttgctggcgtttttccataggctccgcccccctgacgagcatcacaaaaatcgacgct
caagtcagaggtggcgaaacccgacaggactataaagataccaggcgtttccccctggaagctccctcgtgcgctctcct
gttccgaccctgccgcttaccggatacctgtccgcctttctcccttcgggaagcgtggcgctttctcatagctcacgctgtag
gtatctcagttcggtgtaggtcgttcgctccaagctgggctgtgtgcacgaaccccccgttcagcccgaccgctgcgcctta
tccggtaactatcgtcttgagtccaacccggtaagacacgacttatcgccactggcagcagccactggtaacaggattagc
agagcgaggtatgtaggcggtgctacagagttcttgaagtggtggcctaactacggctacactagaaggacagtatttggta
tctgcgctctgctgaagccagttaccttcggaaaaagagttggtagctcttgatccggcaaacaaaccaccgctggtagcg
gtggtttttttgtttgcaagcagcagattacgcgcagaaaaaaaggatctcaagaagatcctttgatcttttctacggggtctga
cgctcagtggaacgaaaactcacgttaagggattttggtcatgagattatcaaaaaggatcttcacctagatccttttaaattaa
aaatgaagttttaaatcaatctaaagtatatatgagtaaacttggtctgacagttaccaatgcttaatcagtgaggcacctatctc
agcgatctgtctatttcgttcatccatagttgcctgactccccgtcgtgtagataactacgatacgggagggcttaccatctgg
ccccagtgctgcaatgataccgcgagacccacgctcaccggctccagatttatcagcaataaaccagccagccggaagg
gccgagcgcagaagtggtcctgcaactttatccgcctccatccagtctattaattgttgccgggaagctagagtaagtagttc

gccagttaatagtttgcgcaacgttgttgccattgctacaggcatcgtggtgtcacgctcgtcgtttggtatggcttcattcagc
tccggttcccaacgatcaaggcgagttacatgatcccccatgttgtgcaaaaaagcggttagctccttcggtcctccgatcgt
tgtcagaagtaagttggccgcagtgttatcactcatggttatggcagcactgcataattctcttactgtcatgccatccgtaaga
tgcttttctgtgactggtgagtactcaaccaagtcattctgagaatagtgtatgcggcgaccgagttgctcttgcccggcgtca
atacgggataataccgcgccacatagcagaactttaaaagtgctcatcattggaaaacgttcttcggggcgaaaactctcaa
ggatcttaccgctgttgagatccagttcgatgtaacccactcgtgcacccaactgatcttcagcatcttttactttcaccagcgtt
tctgggtgagcaaaaacaggaaggcaaaatgccgcaaaaaagggaataagggcgacacggaaatgttgaatactcatac
tcttcctttttcaatattattgaagcatttatcagggttattgtctcatgagcggatacatatttgaatgtatttagaaaaataaacaa
ataggggttccgcgcacatttccccgaaaagtgccacctgacgtctaagaaaccattattatcatgacattaacctataaaaat
aggcgtatcacgaggccctttcgtctcgcgcgtttcggtgatgacggtgaaaacctctgacacatgcagctcccggagacg
gtcacagcttgtctgtaagcggatgccgggagcagacaagcccgtcagggcgcgtcagcgggtgttggcgggtgtcgg
ggctggcttaactatgcggcatcagagcagattgtactgagagtgcaccatatgcggtgtgaaataccgcacagatgcgta
aggagaaaataccgcatcaggcgccattcgccattcaggctgcgcaactgttgggaagggcgatcggtgcgggcctcttc
gctattacgccagctggcgaaagggggatgtgctgcaaggcgattaagttgggtaacgccagggttttcccagtcacgac
gttgtaaaacgacggccagtgccaagctg

Supplementary Figure 15. Lentiviral vector sequence for library construction. Lentivirus LTR sequences were underlined; CMV promoter was shown in red; mouse U6 promoter was shown in blue; filler sequence was shown in grey; GFP sequence was shown in green; P2A sequence was shown in yellow; puromycin resistance gene was shown in purple; PmlI and PmeI restriction sites were highlight in yellow.

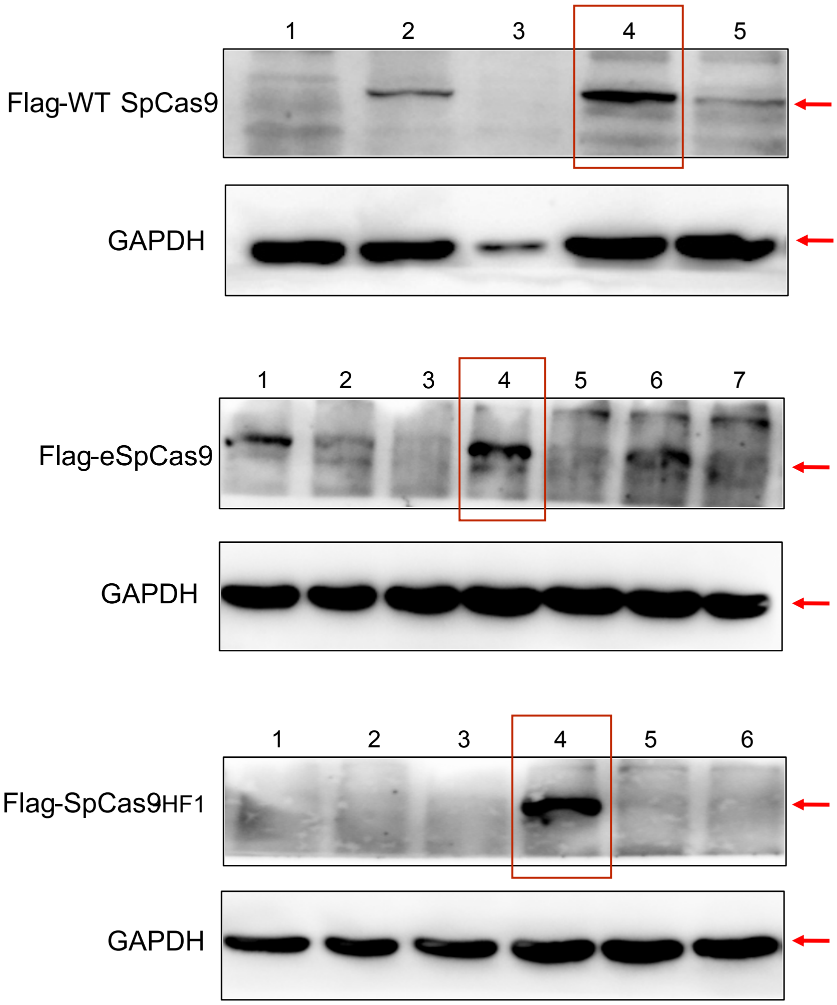

Supplementary Figure 16. The expression of the Cas9 nucleases was confirmed by Western blot. The expected bands were indicated by red arrows; the selected clones in this study were indicated by red box.
