# Supplementary material for: Optimized CRISPR guide RNA design for two high-fidelity Cas9 variants by deep learning
Source: Nat Commun. 2019 Sep 19;10:4284. doi: 10.1038/s41467-019-12281-8 (PMC6753114; doi:10.1038/s41467-019-12281-8)
Supplement: Supplementary file 23 — Description of Additional Supplementary Files [file 41467_2019_12281_MOESM23_ESM.pdf]

Description of Additional Supplementary Data Files

File Name: Supplementary Data 1
Description: The list of 80,263 oligonucleotides that contain gRNAs and corresponding target sequences.

File Name: Supplementary Data 2
Description: gRNA indel rates of 55,604 (covering 20,211 genes), 58,167 (covering 20,315 genes) and 56,888 (covering 20,270 genes) for WT-SpCas9, eSpCas9(1.1) and SpCas9-HF1, respectively.

File Name: Supplementary Data 3
Description: Summary of all important feature information.

File Name: Supplementary Data 4
Description: Tree SHAP values for three Cas9 nucleases.

File Name: Supplementary Data 5
Description: Comparison of the log-odds score for three Cas9 nucleases.

File Name: Supplementary Data 6
Description: Performance comparison of different prediction models for WT SpCas9.

File Name: Supplementary Data 7
Description: Performance comparison of different prediction models for eSpCas9.

File Name: Supplementary Data 8
Description: Performance comparison of different prediction models for SpCas9-HF1.

File Name: Supplementary Data 9
Description: The 100 gRNAs rates of integrated targets, endogenous targets and the prediction models for WT-SpCas9, eSpCas9(1.1) and SpCas9-HF1, respectively.

File Name: Supplementary Data 10
Description: U6 expression transfer Learning without chromatin openness information and T7 expression transfer learning with chromatin openness information.

File Name: Supplementary Data 11
Description: U6 expression transfer learning with chromatin openness information.

File Name: Supplementary Data 12
Description: The performance comparison before and after incorporating the chromosome accessibility feature.

File Name: Supplementary Data 13

Description: The contribution of repetitive nucleotides to gRNA activity.

File Name: Supplementary Data 14
Description: The hyperparameters for XGBoost regression.

File Name: Supplementary Data 15
Description: The hyperparameters for MLP.

File Name: Supplementary Data 16
Description: The hyperparameters for CNN.

File Name: Supplementary Data 17
Description: The hyperparameters for RNN only.

File Name: Supplementary Data 18
Description: The hyperparameters for DeepHF.

File Name: Supplementary Data 19
Description: The raw read counts of the study.

File Name: Supplementary Data 20
Description: The primers sequence used in this study.
